# Critical evaluation of two neutron structures of Mn superoxide dismutase with quantum refinement

**DOI:** 10.1007/s00775-026-02140-5

**Published:** 2026-03-10

**Authors:** Kristoffer J. M. Lundgren, Justin Bergmann, Esko Oksanen, Ulf Ryde

**Affiliations:** 1 Division of Computational Chemistry, Lund University, Chemical Centre, P. O. Box 124, Lund, SE-221 00 Sweden; 2 European Spallation Source ESS ERIC, P. O. Box 176, Lund, SE-221 00 Sweden

**Keywords:** Mn superoxide dismutase, neutron structures, quantum refinement, deprotonated glutamine, deprotonated tyrosine, manganese

## Abstract

In 2021, neutron structures of oxidised and reduced human manganese superoxide dismutase were published, suggesting several unexpected features, including deprotonated glutamate, tyrosine and histidine residues and a reduced Mn ion with two hydroxide ligands. We have used quantum refinement to evaluate whether alternative interpretations of the structures are possible, comparing many structural models with different possible protonation states or other structural interpretations. Quantum refinement is standard crystallographic refinement in which the empirical restraints, which are employed to ensure that the structure makes chemical sense and gives reasonable bond lengths and angles, are replaced by quantum mechanical calculations for a small but interesting part of the structure. We show that in all cases, there are more chemically reasonable interpretations of the structures, not involving any deprotonated residues, which give slightly improved structures in terms of real-space Z scores based on the difference maps and strain energies. Weak nuclear densities do not necessarily indicate that an atom is not present; instead it may indicate that the atom may have several conformations or shows extensive dynamics. For the Tyr-34 residue, our results indicate that the phenolic hydroxide H atom should preferably be within the aromatic ring plane (by ~20 kJ/mol), but with two possible conformations. Moreover, an Mn-bound water molecule can readily accept a hydrogen bond from the nearby Gln-143 residue.

**Supplementary Information:**

The online version contains supplementary material available at 10.1007/s00775-026-02140-5.

## Introduction

Atomistic structural data is essential for the understanding of the function and mechanisms of biological macromolecules, opening for the possibility to manipulate their function by favourable mutations or the design of improved drug candidates. Traditionally, such data has been obtained by X-ray crystallography [23], but over the latest decade, cryogenic electron microscopy has become a complementary alternative [13]. Unfortunately, hydrogen (H) atoms are not discerned in such structures at resolutions typical for biomacromolecules. This is problematic, because they constitute approximately half of the atoms in the structures, and they are essential for the function of the macromolecules and to settle protonation and tautomeric states, as well as the direction of hydrogen bonds.

Fortunately, the position of H atoms can be determined by neutron crystallography [8, 27]. The nuclear scattering length of the ^2^H (deuterium) isotope of H is similar to that of carbon, nitrogen and oxygen, meaning that deuterium positions can be determined in neutron-scattering experiments if all or solvent-exposed H atoms are replaced by deuterium. Neutron crystallography requires very large crystals and long exposure time. Therefore, only ~250 biomolecular neutron structures have been solved, ~1000 times less than the number of X-ray structures. Moreover, it is not always simple to interpret neutron structures, because the data quality is too low or atoms may show dynamics or multiple conformations.

Quantum refinement (QR) is a powerful approach to interpret various types of experimental structural data [6, 26]. Empirical restraints are employed in all biomacromolecular structural methods to supplement the experimental data and ensure that chemically reasonable structures are obtained [14, 15, 20]. In the quantum refinement approach they are replaced with more accurate quantum mechanical (QM) calculations for a small but interesting part of the structure. Thereby, different putative interpretations of the structure can be tested and it can be determined which one fits the experimental data best using both experimental quality measures, like difference maps and real-space Z-scores (RSZD) [30], and QM measures that estimate how much the structure of the QM region changes compared to the ideal QM structure in terms of energies (strain energies) or structure [6, 18]. Previous studies have shown that QR may improve structures [25], determine protonation states of metal ligands [22, 12,5], tautomeric states of drug candidates [9] and discriminate between different interpretations of a structure [5, 11].

Superoxide dismutase (SOD; EC 1.15.1.1) is an enzyme that catalyses the disproportionation of two molecules of the toxic superoxide radical (O_2_^–•^) to molecular oxygen (O_2_) and hydrogen peroxide (H_2_O_2_) [31]. There are several different types of SODs, differing in their metal content (Mn, Fe, Ni or Cu and Zn). Manganese SOD is present in mitochondria and is responsible for the detoxification of oxidative species produced in the electron transport chain [3]. In 2021, Borgstahl and coworkers published neutron structures of both oxidised (Mn^3+^) and reduced (Mn^2+^) human MnSOD at 2.2–2.3 Å resolution [2]. They showed a number of interesting features, e.g. deprotonated glutamine (Gln), histidine (His) and tyrosine (Tyr) residues, as well as two OH^–^ ligands of Mn in the reduced state, with extensive differences observed between the two subunits of the dimeric enzyme.

Considering that several of these features may have strong implications for the reaction mechanism of the enzyme, we here perform QR on the two neutron structures with the aim to decide whether the original suggestions are valid or if there are alternative interpretations that are more or equally likely and chemically more reasonable. For each structure and subunit, we go through a number of possible interpretations of the structure and evaluate which fits the experimental data and QM calculations best. For all sites, we suggest alternative interpretations of the structures.

## Methods

### Quantum refinement

All quantum refinements (QR) were performed with the QRef interface [18] between the Phenix crystallographic software (version 1.21.2–5419) [17] and the ORCA QM software (version 6.0.0) [21]. The refinements were based on the fully deuterated neutron structures of oxidised and reduced MnSOD (protein databank ids 7KKS and 7KKW, respectively). Coordinates, occupancies, B factors and structure factors were downloaded from the protein data bank [7], together with the space group, unit-cell parameters, resolution limits, *R* factors and the test set used for the evaluation of the *R*_free_ factor.

Reciprocal-space refinement was performed with *phenix.refine* [1], involving three macrocycles, where each macrocycle consisted of first coordinate refinement, followed by refinement of the individual atomic displacement parameters (ADP). In the coordinate refinement, only coordinates of residues with at least one atom in the QM region were allowed to move. In the ADP refinement, ADPs for all atoms were allowed to change. After that, real-space Z-scores based on the difference maps (RSZD) were calculated by the use of EDSTATS [30].

All QM calculations were performed with the TPSS density-functional theory method [29] and the def2-SV(P) basis set [28]. We employed the DFT-D4 dispersion correction [10]. The QM regions varied for the various subunits and the sites of interest and are described below (and are shown in Figure S1).

Before running the production QR calculations, we need to settle the *w*_x_ scale factors for the two neutron structures [18]. For the reduced structure, we found in a previous investigation that *w*_x_ = 3 is a proper choice (giving an ideal compromise for the strain energies and the RSZD scores) [18]. This is also close to the suggestion by Phenix for standard crystallographic refinement (2.9–3.5 in different macrocycles). For the oxidised structure, we performed a similar scan of different *w*_x_ values (using the protonation state suggested by the deposited structure). The results are shown in Figure S2. This indicated that also for this structure, *w*_x_ = 3 is a reasonable choice. It is similar to what Phenix suggests, ~2.4. The *w*_QM_ weight factor was kept at 7.5 mol/kcal, which was recommended in our previous study [18].

## Result and discussion

In this study, we systematically examine a number of unusual protonation states suggested by two neutron structures of oxidised and reduced MnSOD (at 2.2 and 2.3 Å resolution, respectively) [2]. We employ quantum refinement to decide whether alternative (chemically more reasonable) interpretations of the structures are possible. To simplify the discussion, we will talk about protons and protonation, although what is actually used and seen in the neutron structures are deuterons. Somewhat confusingly, the deposited coordinate files do not always agree with the text in the publication [2]. Our calculations are based on the deposited files, but we note the discrepancies below. There are conspicuous differences between the two chains in the dimeric proteins. Therefore, we will discuss the two chains in separate subsections.

### Oxidised structure, chain A

**Fig. 1:**
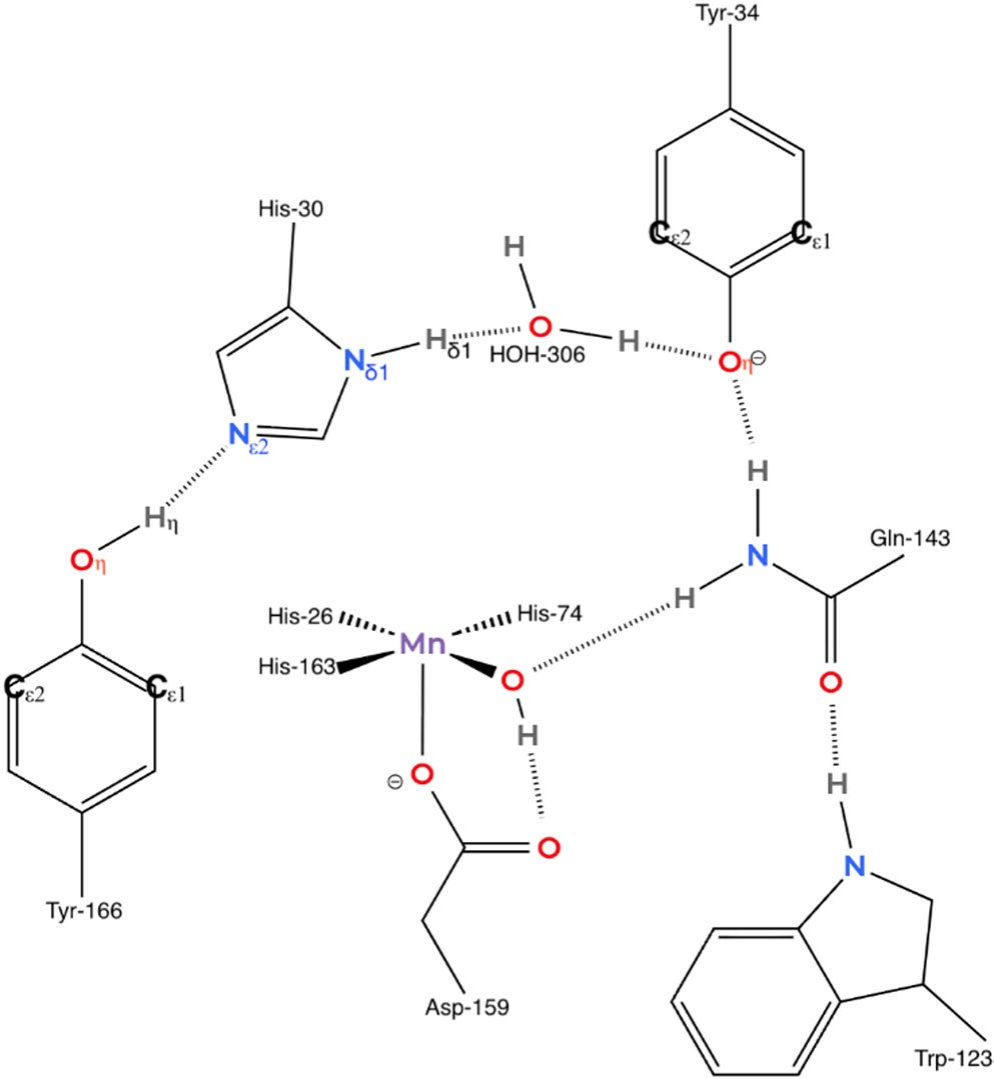
The H-bond network around the active site of oxidised MnSOD. In the deposited structure, the Hη atom on Tyr-166 is missing, as are the two H atoms om Wat-306

In chain A of the deposited structure of oxidised MnSOD, the Mn ion is five-coordinate with three His ligands, a monodentate Asp ligand and a OH^–^ ion [2]. However, two second-sphere Tyr residues, Tyr-34 and Tyr-166 are both deprotonated. The phenolic –OH group of Tyr has a p*K*_a_ of ~ 9.6, so each deprotonated state needs to be stabilised by ~13 kJ/mol at the pH for crystallisation (7.4 [2]). Still, deprotonated Tyr residues are known in several other proteins [4, 16, 19]. The two Tyr residues form a H-bond network running from Tyr-166, via His-30, a water molecule (HOH-306), Tyr-34, Gln-143 and finally to both the Mn-bound solvent molecule and to Trp-123, as is shown in Fig. 1. His-30 is singly protonated on the Nδ1 atom (atom names are also shown in Fig. 1), whereas no protons are reported for HOH-306 in the deposited structure. Hδ1 forms a weak hydrogen bond to HOH-306 (2.22 Å) and the distance between HOH-306 and the phenolic oxygen atom of Tyr-34 (Oη) is only 2.28 Å, without any proton in between. Likewise, no H atom is reported between His-30 and Tyr-166, although the Oη–Nε2 distance is only 2.63 Å. However, according to the publication [2], there should be a proton on Oη of Tyr-166, forming a strong H bond to Nε2 of His-30 (1.7 Å). The other side of Tyr-166 does not contain any H-bond partners and it is partly covered by the non-polar sidechain of Leu-25, but there is also empty space which might contain a water molecule (but none is observed, neither in the neutron or in the corresponding X-ray structure, 7kku at 2.02 Å resolution). HOH-306 is also present in the X-ray structure (HOH-307, at an O–O distance of 0.5 Å between the overlayed structures).

We initially tested ten plausible interpretations of the hydrogen-bond network between Tyr-166, His-30, HOH-306 and Tyr-34. Tyr-166 was either deprotonated (D) or protonated with the proton directed away from (L; to the left in Fig. 1) or towards His-30 (R). His-30 was either protonated on the Nε2 atom (E), on Nδ1 (D), on both N atoms (P) or on neither of them (M). Tyr-34 was either deprotonated (D) or protonated with the proton pointing towards HOH-306 (P). It always receives a H bond from Gln-143 on the other side. It was assumed that HOH-306 always is a water molecule that adapts to the protonation of His-30 and Tyr-34 so that there is an intact H-bond chain and with protons pointing in other directions, if not needed for the H bonds (there are no additional H-bond partners of this molecule in the neutron structure, but there is plenty of space). Consequently, we describe the structures by three letters describing the protonation of Tyr-166, His-30 and Tyr-34, in this order, cf. Table 1 (for example, RDP indicates that Tyr-166 is protonated with the proton pointing towards His-30, His-30 is protonated on the Nδ1 atom and Tyr-34 is protonated with the proton pointing towards HOH-306). A final lower-case letter indicates different conformations of the same H-bond pattern. In five cases, one or more H atoms moved to another residue during the refinement, indicating that the original structure is not stable in the QM calculation. However, we then performed also QR with the protons restrained to be at the original position (the structures in Table 1 always represent the protonation state after QR).

**Table 1:** Net charge of the QM system (ch), strain energies (∆*E*_str_ in kJ/mol), intrinsic QM energies (∆*E*_QM_ in kJ/mol), average RSZD scores of residues in the QM system (the individual values are shown in Table S1), as well as H-bond distances (H–O or H–N distances in Å; HB1 is between Tyr-166 and His-30, HB2 is between His-30 and HOH-306, HB3 is between HOH-306 and Tyr-34 and HB4 is between Tyr-34 and Gln-143) for the deposited structure and the various QR structures of oxidised MnSOD, chain A. The naming of the structures is explained in the text. The upper part of the table contains the initial 16 QR calculations, whereas the lower part contains the subsequent eight QR calculations

Structure	ch	∆*E*_str_	∆*E*_QM_	RSZD	HB1	HB2	HB3	HB4
deposited	-2	214		1.23	1.66	2.21	1.23	2.33
DDD	-2	160	114	1.85	2.11	1.63	1.84	2.79
DEDa	-2	72	4	0.90	2.01	1.85	2.07	1.48
DEDb	-2	68	0	1.03	1.98	1.85	2.07	1.47
DEP	-1	99	37	0.93	2.30	1.49	1.67	1.50
LED	-1	84	161	0.73	2.04	1.79	2.06	1.71
RDD	-1	70	0	0.60	2.17	1.54	1.72	1.64
DPD	-1	109	47	0.85	2.27	1.49	1.65	1.39
RMP	-1	89	23	0.70	2.31	1.44	1.56	1.58
LEP	0	73	0	0.75	2.30	1.51	1.71	1.65
LPD	0	142	75	0.58	2.35	1.33	1.52	1.63
RDPa	0	51	7	0.75	2.26	1.71	2.01	1.69
DPP	0	111	74	0.73	2.31	1.75	1.98	1.30
LPP	1	113	0	0.60	2.32	1.80	1.86	1.55
RMD	-2	104	36	0.68	1.99	1.86	2.05	1.58
RM1	-1	156	89	0.68	2.16	2.12	1.85	1.53
RM2	-1	172	106	0.73	2.38	2.14	1.78	1.56
RDPb	0	69	26	0.65	2.24	1.74	2.13	1.71
RDPc	0	56	13	0.63	2.20	1.77	1.92	1.70
RD1a	0	46	3	0.70	2.40	1.78	1.80	1.68
RD1b	0	57	14	0.55	2.41	1.81	1.90	1.70
RD2	0	50	7	0.73	2.66	1.76	1.81	1.70

The results of 13 quantum refinements are collected in the upper part of Tables 1 and S1 (two of the refinements ended up in identical structures). The structures are judged by two quality measures. RSZD shows how well each residue in the QM region fits the neutron scattering map, i.e. how well the model fits the experimental data. The strain energy (∆*E*_str_) measures how much the QM region in the QR structure differs from the ideal QM structure. The ideal QM structure is taken as the best geometry-optimised structure with the same H-bonds. For each net charge of the QM region (ch in Table 1), we also report the relative QM energy of each QR structure (∆*E*_QM_, i.e. the intrinsic stability of the QM region compared to the same region in the other QR structures). The net charge depends on the total number of protons in the QM region (the H-bond chain) and it varies from − 2 to + 1.

From Table S1, it can be seen that the RSZD of Gln-143 is essentially the same (0.1–0.2) in all QR structures, reflecting that it has the same protonation state in all structures. Somewhat unexpectedly, the RSZD of Tyr-34 also shows only a minimal variation, 0.9–1.2, and with no correlation to its protonation state. Apparently, the fit of the model to the experimental data is indifferent to the protonation state of this residue.

On the other hand, the RSZD of Tyr-166 shows a large variation (0.7–3.7). The worst result is obtained for the protonation state present in the deposited structure (DDD, indicating that Tyr-166 is deprotonated, His-30 is protonated on the Nδ1 atom and Tyr-34 is deprotonated; but in variance to the deposited structure, two protons were added to the water molecule, one forming a H bond to Tyr-34). This structure is much worse than all the other structures and will not be further discussed. Among the other structures, the RSZD of Tyr-166 is highest if Tyr-166 is deprotonated, 1.2–1.6, it is intermediate if it is protonated with the proton pointing away from His-30 (L), 0.8–1.0, whereas it is best if the proton on Tyr-166 points towards His-30 (R), 0.7.

The RSZD of His-30 also shows quite some variation, 0.4–1.1. Unfortunately, there is little correlation to the protonation state of the residue: The three lowest values are all obtained for the doubly protonated (HIP) state, but a fourth HIP structure has a RSZD value of 0.9. Likewise, four Nε2 protonated structures (HIE) have high RSZD values (1.0–1.1), but the fourth has 0.7. The two Nδ1 protonated (HID) structures have RSZD values of 0.6 and 1.1.

The RSZD of HOH-306 are mostly 2.1–2.4. However, it is lower for two structures, 1.6 for LED and 1.7–1.8 for DED. These are the only two structures in which the water molecule donates hydrogen bonds to both Nδ1 of His-30 and Oη of Tyr-34, and all three atoms of the water molecule are in nearly identical positions in the two structures.

Taking the average of the RSZDs of the five residues in the QM region gives nearly the same results for most QR structures, 0.6–0.9 (Table 1). The best results are obtained for the RDD, LPD and LPP structures.

The strain energies vary from 50 to 160 kJ/mol. The best result is obtained for the RDP structure, which is also chemically the most reasonable structure, not involving any unusual protonation state of any residue. However, it has a relatively large average RSZD score, 0.75. Moreover, in this structure, the water molecule is not involved in any hydrogen bonds, which makes it intrinsically less stable than the LEP structure by 7 kJ/mol (∆*E*_QM_ in Table 1). The DED structures have the lowest strain energy among the structures with a net charge of − 2 in the QM region, 68–72 kJ/mol, but they have a large average RSZD score, 0.9–1.0. RDD has the lowest strain energy for the structures with a −1 net charge of the QM region, 70 kJ/mol. It also has a low average RSZD, 0.60. It is the structure suggested in the original article (but not the one shown in their schematic Fig. 6) [2]. On the other hand, the RDP structure with a water molecule not involved in any H bonds may explain why no H atoms are observed in the original neutron structure: They may simply move rather freely, attaining many different conformations. It may also explain why HOH-306 is seen at a lower density level (1.6 σ) than the other residues in the QM region (3.2–6.4 σ).

A problem with all QR structures with a protonated Tyr-34 is that the proton points towards the water molecule and therefore is out of the phenolic plane, which has a barrier of ~20 kJ/mol (cf. Figure S2). A possible solution is that Tyr-34 is protonated with the proton within the phenolic plane and therefore not forming any H bond to HOH-306 (which instead donates a H bond to Oη). The proton on Tyr-34 could then be in two different positions (pointing towards either CE1 or CE2 in the phenol ring ; these two conformations are called 1 or 2 in the following). If both positions are partly occupied in the structure, this would weaken their densities and may explain why it is not seen in the structure.

Another problem is that RSZD of HOH-306 indicates that it should donate hydrogen bonds to both His-30 and Tyr-34. Together with the fact that Tyr-166 should be protonated with the proton pointing towards His-30, it indicates that His-30 should be doubly deprotonated (and therefore negatively charged; HIM).

Based on these three considerations, we constructed nine new structures. Three of them involve a doubly deprotonated His-30 (and Tyr-166 protonated towards His-30). They differ in whether Tyr-34 is deprotonated (RMD) or protonated with the proton within the phenol ring plane in the two possible directions (RM1 and RM2). From the results in Table 1, it can be seen that none of them were especially successful in terms of strain energies and RSZD scores (besides that RM2 gives the best RSZD score of HOH-306, 1.4).

**Fig. 2:**
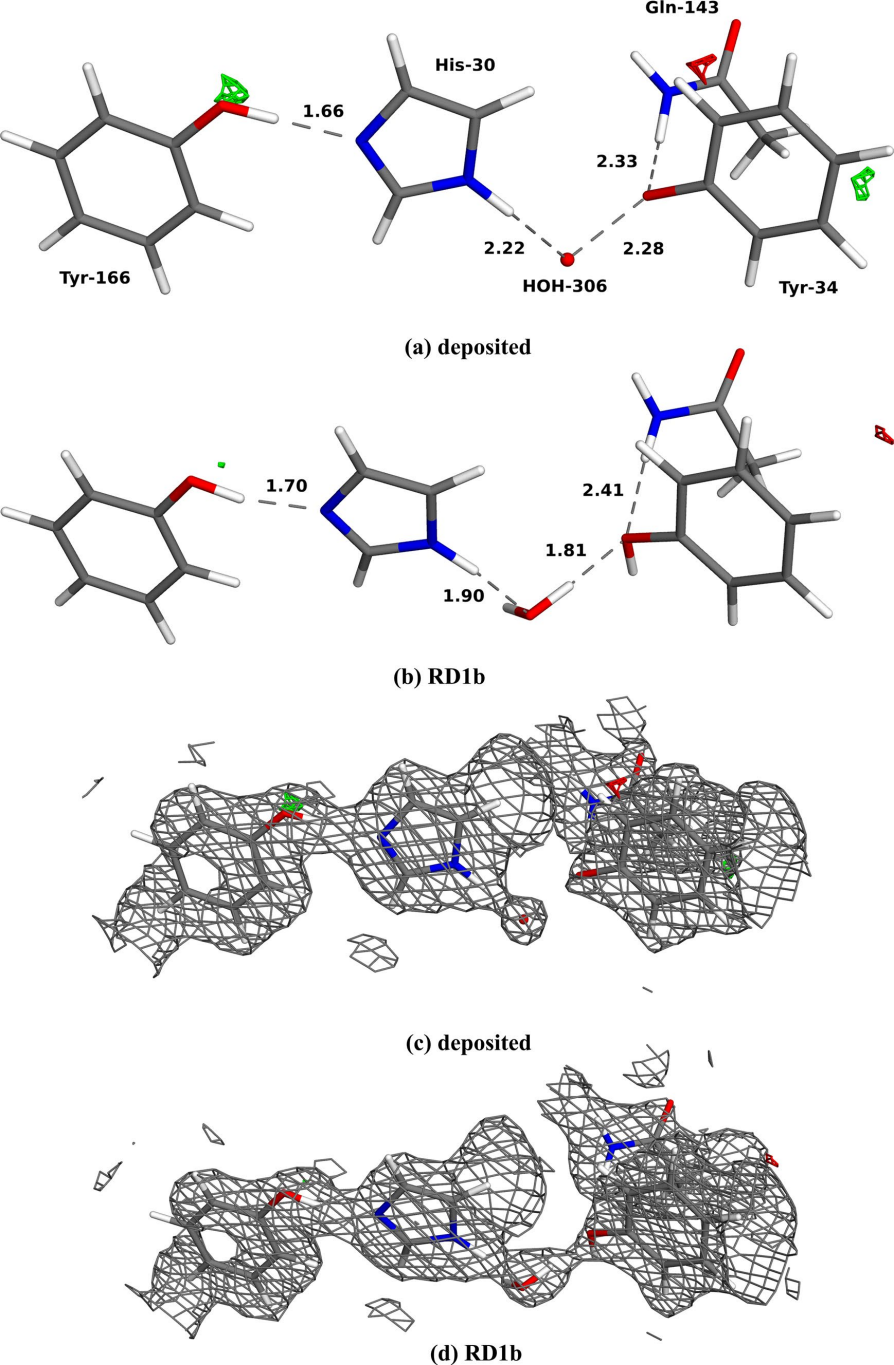
Deposited structure (RDD state) (**a**) and QR structure (RD1b state) (**b**) of oxidised MnSOD, chain A. In the deposited structure, a proton has been added according to the suggestions in the publication (Azadmanesh et al., 2021). Hydrogen bond lengths are shown in Å. The *mF*_o_ – *DF*_c_ difference maps are contoured at +3 σ (green) and –3 σ (red). (**c**) and (**d**) show the same two structures with the 2*mF*_o_ – *DF*_c_ map contoured at +1 σ (grey)

The other six structures are based on the RDP model, but involve either rotation of the HOH-306 molecule (one H atom forms a H bond to Tyr-34, but the other does not interact with any atom in the QM region) or movement of the proton on Tyr-34 into the phenol plane with the two possible directions (RD1 or RD2). Interestingly, one of the rotated RDP structures converged into a RD2 structure instead (not included in Table 1). One of the rotated RDP structures gives the lowest RSZD score for Tyr-166 (0.5) and also the second-lowest RSZD for HOH-306 (1.5), showing that also structures with HOH-306 donating only one hydrogen bond may give a low RSZD score for HOH-306. One of the RD1 structures gives the lowest average RSZD score of all structures (0.55) and the second-lowest RSZD of His-30 (Fig. 2b). The other RD1 structure gives a quite low RSZD score for HOH-306, 1.8. These results indicate that the variations in the RSZD scores are close to the noise level.

The strain energies of these five structures are also among the lowest of all structures, 46–69 kJ/mol. Therefore, we would suggest RD1 as our best interpretation of the neutron structure, although the results indicate that the structure is flexible and several conformations may be populated. Figures 2c and d show that the neutron scattering-length density gives a good support to the HOH-306 H atom pointing towards Tyr-34, even if it was not visible in the deposited structure, whereas the other H atom is less supported, indicating that it is more flexible (as is illustrated by the other RD1 structure, which differs mainly in the position of this atom). There is some support for the HO proton on Tyr-34, but it can probably also partly be in the other direction or move more or less out of the phenol plane, as is suggested by the RDP structures. The average RSZD score is much better than for the deposited structure (even after addition of the missing Hη atom on Tyr-166), 0.55 compared to 1.2. The strain energy is also much lower, 57, compared to 214 kJ/mol.

We do not claim that this RD1b structure fits the data much better than the RDD structure, suggested in the original study. Undoubtedly, the H atoms on HOH-306 and HO on Tyr-34 are weakly supported by the experimental data (in fact, HOH-306 itself is rather weakly supported), but this does not prove that they are not there. A more plausible interpretation is that they show disorder or increased dynamics. Thus, we argue that there is no strong support for the suggestion that Tyr-34 is deprotonated in the oxidised state.

### Oxidised structure, chain B

**Fig. 3:**
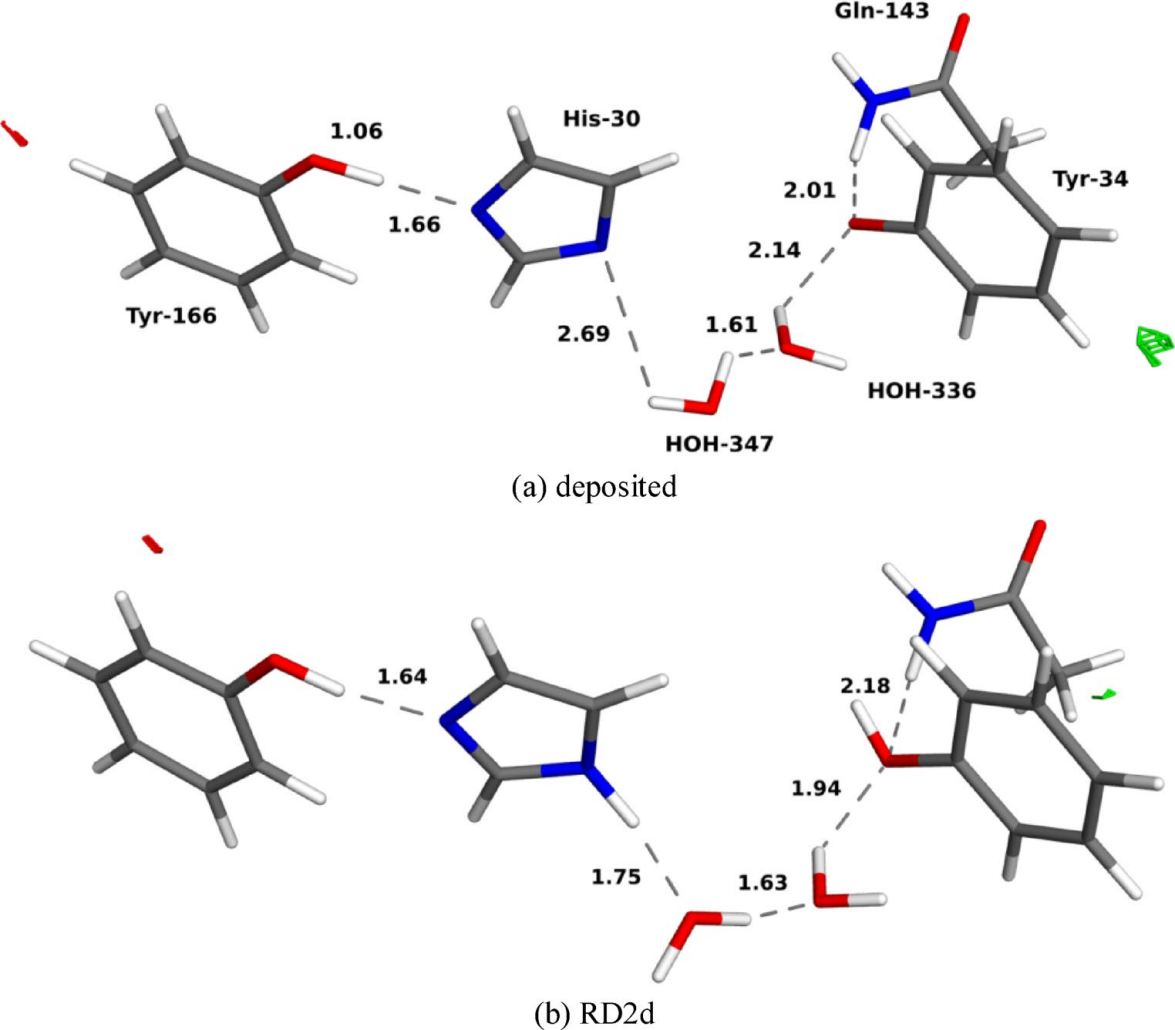
Deposited structure (RMD state) (**a**) and QR structure (RD2d state) (**b**) of oxidised MnSOD, chain B. In the deposited structure, a proton has been added according to the suggestions in the publication (Azadmanesh et al., 2021). Hydrogen bond lengths are shown in Å. The *mF*_o_ – *DF*_c_ difference maps are contoured at +3 σ (green) and –3 σ (red).

The corresponding active-site structure in chain B is quite similar. Mn is still five-coordinate with a OH^–^ ion and both Tyr-34 and 166 are still deprotonated in the deposited structure, although Tyr-166 is protonated according to the article [2]. However, His-30 is modelled as a doubly deprotonated imidazolate sidechain (Fig. 3a). The latter is quite unlikely as the corresponding p*K*_a_ is ~ 14. The distance between the Oη atom of Tyr-34 and the Nδ1 atom of His-30 has increased from 4.65 Å in chain A to 5.30 Å in chain B and there are two, rather than one water molecule between these two atoms. One (HOH-336) is 2.14 Å (H–O) from Tyr-34 and another (HOH-347) is 2.69 Å (H–O) from Nδ1 of His-30. They are connected by a H bond (1.61 Å H–O distance), but the geometries are far from ideal for all three hydrogen bonds. Neither of the water molecules is supported by the corresponding X-ray structure, which does not show any water molecule between His-30 and Tyr-34 (the Nδ1–Oη distance is 5.04 Å). This strongly indicates that the His-30 and Tyr residues are not deprotonated, because charged residues normally form very strong hydrogen bonds.

We ran QR for 20 different interpretations of the protonation state. The results are gathered in Tables 2 and S2. The RSZD of Gln-143 varies somewhat more than in chain A, 0.9–1.4. It is lowest for some of the RD1 and RD2 structures, but since the protonation state of this residue does not vary, it is hard to make any interpretation. The RSZD of Tyr-34 shows a rather small variation (0.1–0.6) and the various protonation states all show similar ranges. The same applies to the RSZD scores of Tyr-166, 0.4–1.2.

**Table 2:** Net charge of the QM system (ch), strain energies (∆*E*_str_ in kJ/mol), intrinsic QM energies (∆*E*_QM_ in kJ/mol), average RSZD scores of residues in the QM system (the individual values are shown in Table S2), as well as H-bond distances (H–O or H–N distances in Å; HB1 is between Tyr-166 and His-30, HB2 is between His-30 and HOH-347, HB3 is between HOH-347 and HOH-336, HB4 is between HOH-336 and Tyr-34 and HB5 is between Tyr-34 and Gln-143) for the deposited structure and the various QR structures of oxidised MnSOD, chain B.

Structure	ch	∆*E*_str_	∆*E*_QM_	RSZD	HB1	HB2	HB3	HB4	HB5
deposited	-2	342	0	0.95	1.66	2.69	1.62	2.72	2.01
DMD	-3	285	0	0.75	2.92	2.19	1.80	2.16	1.61
DEDa	-2	72	0	0.80	1.50	2.22	1.76	1.86	1.69
DEDb	-2	96	7	0.75	1.48	2.39	1.76	1.87	1.69
RMD	-2	105	31	0.70	1.55	2.25	1.76	1.90	1.67
DEP	-1	121	32	0.77	1.49	1.69	1.52	1.58	1.86
LED	-1	105	141	0.78	1.72	2.23	1.74	1.84	1.69
RDD	-1	89	0	0.68	1.61	1.75	1.57	1.72	1.75
RMP	-1	106	18	0.67	1.50	1.61	1.50	1.57	1.85
LEP	0	92	2	0.88	1.68	1.83	1.59	1.62	1.87
RDP	0	100	15	0.77	1.68	2.31	1.70	1.77	1.86
RD1a	0	98	13	0.67	1.65	1.86	1.65	2.06	1.94
RD1b	0	108	24	0.92	1.66	1.88	1.82	1.94	1.95
RD1c	0	111	26	0.98	1.66	1.96	1.83	1.89	1.94
RD1d	0	91	7	0.73	1.65	1.78	1.68	2.19	1.95
RD1e	0	114	30	0.83	1.66	1.83	1.68	1.99	1.98
RD2a	0	86	2	0.68	1.65	1.73	1.61	1.87	2.22
RD2b	0	108	24	0.88	1.66	1.91	1.77	1.78	2.16
RD2c	0	115	30	0.83	1.66	2.11	1.80	1.83	2.13
RD2d	0	84	0	0.65	1.64	1.74	1.63	1.94	2.18
RD2e	0	112	28	0.77	1.66	1.81	1.67	1.95	2.10

The RSZD scores of His-30 show a similar variation, 0.6–1.3, but with a better correlation to the structures. The HIE state always give the largest values (1.0–1.3), whereas HID (0.7–0.9) and HIM (0.6–0.9) give lower values.

The RSZD scores of HOH-336 are in general 0.4–0.8 when it donates a hydrogen bond to the other water molecule and receives a hydrogen bond from a protonated Tyr-34. But in some cases, much larger values are obtained, 1.2–2.3, showing that the RSZD depends strongly on the position of the H atom that does not form any H bonds (the various RD1 and RD2 structures sample different positions of the water protons). If HOH-336 donates a H bond to a deprotonated Tyr-34 (and receives a hydrogen bond from the other water molecule), RSZD is slightly larger and more consistent, 0.8–0.9.

The RSZD score of the other water molecule (HOH-347) is largest if it receives hydrogen bonds from both His-30 and HOH-336, 1.3. It is intermediate (0.8–1.1) if it donates a hydrogen bond to His-30 and receives a hydrogen bond to HOH-336 (0.8–1.1) and is lower if it donates hydrogen bonds both to His-30 and HOH-336 (0.5–0.6). However if it receives a hydrogen bond from His-30 and donates a hydrogen bond to HOH-336, it can attain a wide range of RSZD values, from 0.4 to 1.2, showing that the score depends strongly on the direction of the H atom that does not form any H bond. It is somewhat questionable how much trust can be put to these two water molecules that are not supported by the X-ray data and show a different structure in the other subunit.

The average RSZD scores of the six residues in the QM region show only a small variation among the nine tested structures, 0.65–0.98, which may be within the noise level. The structure giving the best results is one of the RD2 structures.

The DED structure, with a net charge of −2 in the QM region, has the lowest strain energy among the various structures, 72 kJ/mol. However, it gives a rather high average RSZD score (0.80), especially for His-30 (1.2). RDD gives the lowest strain energy among the structures with a net −1 charge of the QM region, 89 kJ/mol, partly reflecting that each of the two water molecules donates only one H bond each (in the DED structure, HOH-336 donates two H bonds). It gives a better average RSZD score, 0.68. A RD2 structure (RD2d with neutral QM regions) has a quite low strain energy, 84 kJ/mol (again the two water molecules donate one H bond each). It has the intrinsically lowest energy among the neutral structures and also the lowest average RSZD score among all structures (0.65). Therefore, this is our best interpretation of this site. Again, it seems likely that both conformations of the Hη atom of Tyr-34 (RD1 and RD2) are populated. The structure is shown in Fig. 3b and it can be seen that there are no significant features in the difference map. This indicates that there is no experimental support for any deprotonated Tyr or His residues in this structure. The two water molecules between Tyr-34 and His-30 are not supported by the X-ray structure and only one water is discerned in subunit A. Thus, this part of the structure may be quite flexible. The average RSZD score of our best structure is better than for the deposited structure (even after addition of the missing Oη atom on Tyr-166), 0.65 compared to 0.95. The strain energy is also much lower, 84 kJ/mol, compared to 342 kJ/mol.

### Reduced structure, chain B

**Fig. 4:**
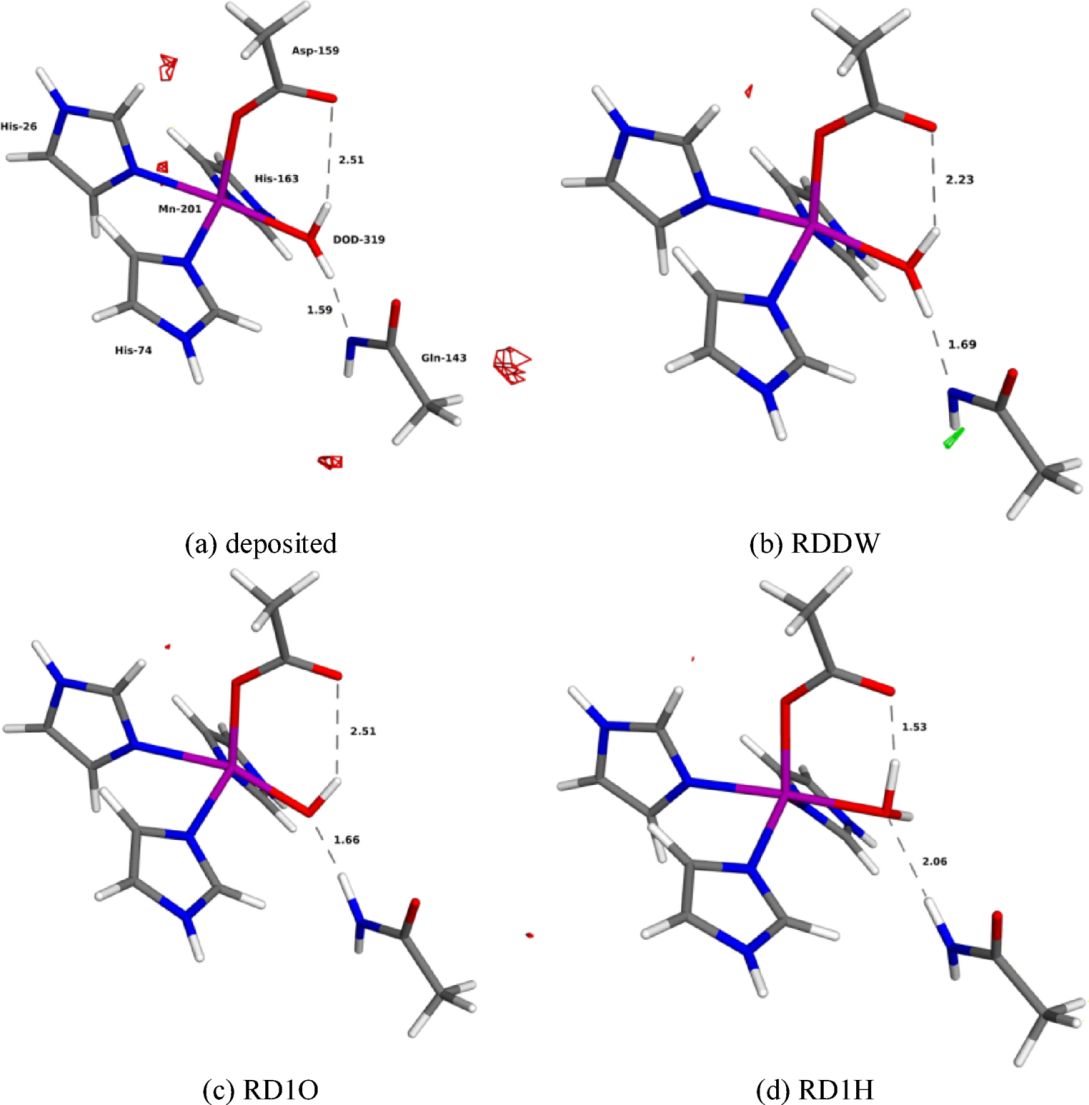
Deposited structure (RDDW state) (**a**) and three QR structures with (**b**) a deprotonated Gln-143 and water (RDDW state), (**c**) a protonated Gln-143 and OH^–^ (RD1O state) and (**d**) a protonated Gln-143 and water for the Mn site of reduced MnSOD (RD1H state), chain B, concentrated on the Mn site. Hydrogen bond lengths are shown in Å. The *mF*_o_ – *DF*_c_ difference maps are contoured at +3 σ (green) and –3 σ (red)

**Fig. 5:**
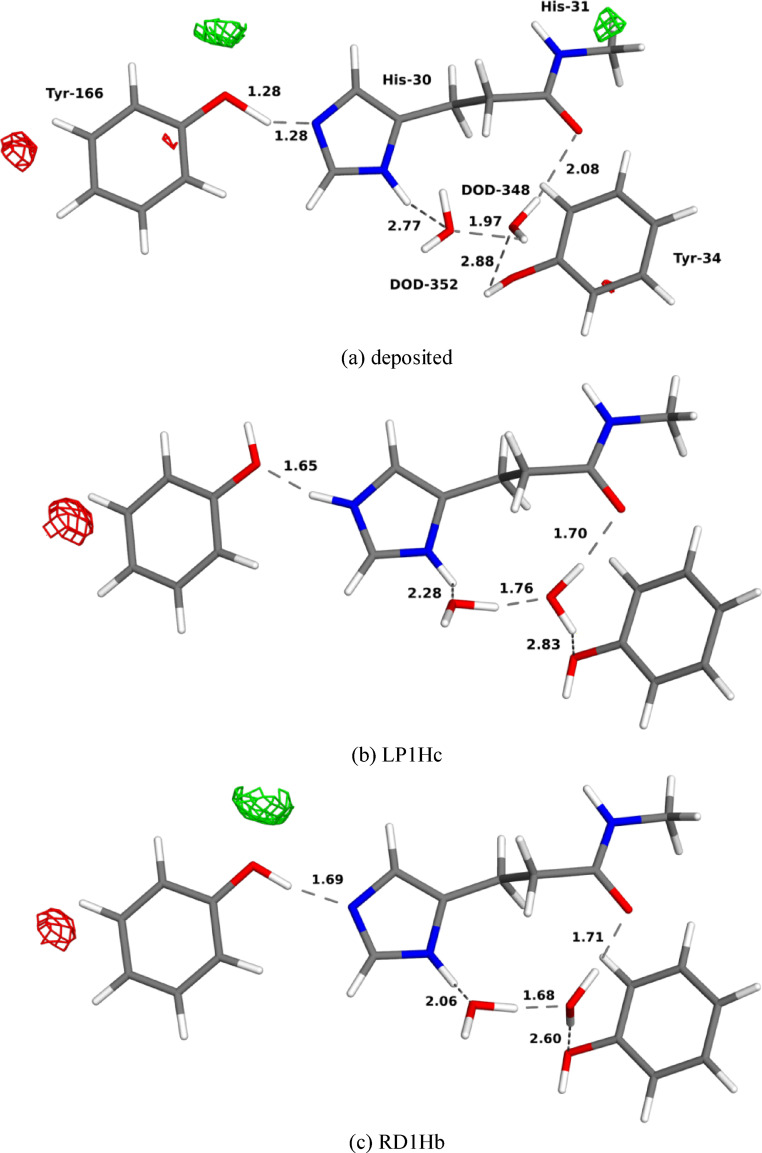
Deposited structure (RDP state) (**a**) and two QR structures in the LP1Hc (**b**) and RD1Hb states (**c**) of the H-bond chain in reduced MnSOD, chain B. Hydrogen bond lengths are shown in Å. The *mF*_o_ – *DF*_c_ difference maps are contoured at +3σ (green) and –3σ (red).

Next, we turned to the reduced MnSOD structure. We start with chain B, which still has a five-coordinate Mn ion. As expected, the solvent-derived Mn-ligand is water [24]. However, it donates a hydrogen bond to Gln-143, which is deprotonated (Fig. 4a). This is quite unexpected, because the p*K*_a_ of acetamide (a simple model of the Gln sidechain) is 15.1, much higher than water in Mn^2+^(H_2_O)_6_ (~10.6). Tyr-34 is protonated with the proton out of the phenol plane (with a long H–O bond length of 1.29 Å) and His-30 is protonated on Nδ1 (Fig. 5a). However, Tyr-166 shares a proton with His-30 (both O/N–H distances are 1.28 Å in the deposited structure, but they are reported as 1.3 and 1.2 Å in the publication [2]. There is a water molecule (DOD-348) that accepts very long H bonds from both Tyr-34 and Nδ1 of His-30, with 2.88 and 2.92 Å H–O/N distances. It is present also in the corresponding X-ray structure. It donates a H bond to the backbone O atom of His-30. There is another water molecule in the neutron structure (DOD-352), which is not seen in the corresponding X-ray structure (7klb at 2.16 Å resolution). It is only 2.20 Å away (O–O distance) from DOD-348, but they are not connected with any H bonds. Thus, our main question is whether Gln-143 is really deprotonated, but we also look at the hydrogen-bond network from Gln-143 via Tyr-34, water and His-30 to Tyr-166. In particular, we tested to move the proton on Tyr-34 into the ring plane and let the water molecule donate a H bond to it.

Consequently, we constructed a large QM region, including both the Mn site, as well as the hydrogen-bond chain from the Mn-ligand, via Tyr-34, DOD-348 and His-30 to Tyr-166. We included also DOD-352, Trp-123 and the backbone of His-30 (including a –NHCH_3_ cap from His-31; shown in Figure S1c). For Gln-143 and the Mn-bound solvent molecule (DOD-319), we tested three different conformations: Gln-143 deprotonated and a water molecule (called W), Gln-143 protonated and OH^–^ (O) or Gln-143 protonated and water (H; Figs. 5b–d). We also tried different directions of the protons on the two water molecules (final letter a, b and c). Therefore, the structures are described by four or five letters (three for Tyr-166, His-30 and Tyr-34 as usual, the fourth for Gln-143/DOD-319 and the last for the conformation of water protons). We optimised 29 different structures, described in Tables 3 and S3.

**Table 3:** Net charge of the QM system (ch), strain energies (∆*E*_str_ in kJ/mol), intrinsic QM energies (∆*E*_QM_ in kJ/mol), average RSZD scores of residues in the QM system (the individual values are shown in Table S3), as well as H-bond distances (H–O or H–N distances in Å; HB1 is between Tyr-166 and His-30, HB2 is between His-30 and DOD-348, HB3 is between DOD-348 and Tyr-34, HB4 is between Tyr-34 and Gln-143, HB5 is between Gln-143 and DOD319, HB6 is between DOD-319 and Asp-159, HB7 is between Gln-149 and Trp-123, HB8 is between DOD-352 and the backbone O of His-30, and HB9 is between DOD-348 and DOD-352) for the deposited structure and the various QR structures of reduced MnSOD, chain B

Structure	ch	∆*E*_str_	∆*E*_QM_	RSZD	HB1	HB2	HB3	HB4	HB5	HB6	HB7	HB8	HB9
deposited	0	706	552	1.24	1.28	2.77	2.88	1.70	1.59	2.51	1.48	2.08	1.97
DPDW	0	289	135	0.91	1.33	2.60	2.70	2.17	1.69	2.23	1.83	1.96	2.03
RDDW	0	235	80	0.96	1.68	2.63	2.52	2.20	1.69	2.28	1.64	1.98	1.99
RDPOa	0	181	38	0.99	1.67	2.65	2.63	2.08	1.67	2.46	1.71	1.96	1.99
RDPOb	0	176	33	1.03	1.70	2.64	2.49	2.06	1.69	2.51	1.74	1.97	1.94
RD1Oa	0	155	12	0.86	1.67	2.82	2.52	2.12	1.66	2.50	1.73	1.74	1.75
RD1Ob	0	154	11	0.92	1.67	2.93	2.50	2.13	1.65	2.51	1.74	1.75	1.72
RD1Oc	0	150	7	0.87	1.67	2.90	2.46	2.13	1.65	2.50	1.73	1.75	1.72
RD2Oa	0	145	2	0.86	1.69	2.66	2.82	2.11	1.71	2.55	1.77	1.76	1.80
RD2Ob	0	143	0	0.91	1.68	2.71	2.74	2.10	1.70	2.60	1.76	1.74	1.72
RD2Oc	0	143	0	0.88	1.68	2.71	2.71	2.09	1.71	2.57	1.76	1.75	1.74
LPDW	1	278	230	0.97	1.69	2.35	2.82	2.19	1.68	2.23	1.63	1.99	2.20
LPPOa	1	231	185	0.96	1.68	2.46	2.66	2.07	1.81	2.45	1.74	1.97	2.13
LPPOb	1	337	179	0.95	1.71	2.43	2.68	2.05	1.81	2.50	1.76	1.97	2.10
RDPH	1	206	20	1.01	1.71	2.60	2.45	2.02	2.08	1.63	1.79	1.95	1.94
RD1Ha	1	191	5	0.82	1.70	2.78	2.66	2.02	2.07	1.53	1.79	1.72	1.77
RD1Hb	1	186	0	0.81	1.69	2.94	2.60	2.03	2.06	1.53	1.79	1.71	1.68
RD1Hc	1	197	11	0.82	1.70	2.83	2.57	2.01	2.05	1.60	2.57	1.77	1.72
RD2Ha	1	201	15	0.86	1.70	2.65	3.02	2.01	2.09	1.56	1.81	1.73	1.81
RD2Hb	1	198	12	0.83	1.69	2.67	2.97	2.02	2.09	1.55	1.81	1.71	1.77
RD2Hc	1	207	21	0.84	1.70	2.62	2.97	1.99	2.07	1.61	1.80	1.73	1.81
LE1H	1	205	45	0.90	1.77	2.69	2.32	1.99	2.03	1.67	1.78	1.85	1.96
LE2H	1	230	70	0.90	1.77	2.35	2.75	1.99	2.08	1.60	1.81	1.82	1.89
LPPH	2	260	43	0.96	1.65	2.42	2.67	2.03	2.09	1.62	1.79	1.94	2.13
LP1Ha	2	217	0	0.83	1.64	2.60	2.96	2.02	2.12	1.45	1.81	1.70	1.76
LP1Hb	2	223	6	0.86	1.64	2.63	2.77	2.04	2.05	1.57	1.79	1.69	1.71
LP1Hc	2	219	2	0.81	1.65	2.54	2.83	2.02	2.09	1.50	1.80	1.70	1.76
LP2Ha	2	249	32	0.89	1.64	2.55	2.91	2.01	2.10	1.57	1.82	1.74	1.75
LP2Hb	2	233	16	0.90	1.64	2.58	3.15	2.02	2.15	1.42	1.82	1.69	1.74
LP2Hc	2	242	26	0.87	1.65	2.52	2.96	2.01	2.13	1.52	1.81	1.72	1.79

The RSZD scores of the Mn ligands, as well as of His-31 and Trp-123, show little variation between the various structures (0.1–0.5) with no correlation to the protonation states. However, the RSZD scores of Mn itself are strongly discriminating. Those for structures with a water ligand and a protonated Gln-143 are much lower, 0.5–0.7, than those with water and a deprotonated Gln-143 (1.2–1.4) or with OH^–^ and a protonated Gln-143 (1.3–1.5). This is also supported by the RSZD scores of Gln-143, but to a smaller extent: Structures with a water ligand and a protonated Gln-143 has slightly lower RSZD scores (0.1–0.3) than the other two types of structures (0.3–0.5 and 0.4–0.6, respectively). On the other hand, the RSZD scores of the solvent ligand itself (DOD-319) show overlapping ranges for the three types of structures, 1.0–1.5, 1.0–1.4 and 1.1, respectively. Thus, it seems clear that a water ligand and a protonated Gln-143 is the best interpretation of this part of the structure.

The RSZD of His-30 also varies quite a lot (0.3–2.1). It is in general lowest for HID (0.3–0.7), but five HID structures also give larger scores (1.0–1.2). On the other hand, most HIP structures give rather high scores (0.8–1.2), but one structure gives a low score (0.4, DPDW, possibly because the Nε2–Hε2 bond length is quite long, 1.12 Å, giving it partly HID character). The two HIE structures give large scores, 1.2 and 2.1.

The RSZD values of Tyr-166 is lowest when the proton points away from His-30 (0.5–0.7) and it is higher when the proton points in the opposite direction, 0.8–1.2. For the only deprotonated structure tested, RSZD is intermediate, 0.9. This is a bit inconsistent with the results from His-30, because the lowest scores are obtained when there is no proton between Tyr-166 and His-30.

RSZD of Tyr-34 is always smaller for the protonated structures (0.9–1.4) than for the deprotonated structures (1.5–1.8). The in-plane position of the proton is better than the out-of-plane position, especially when it points towards CE1 (0.9–1.1, vs. 1.1–1.4; 1.1–1.3 when it points towards CE2). However, it should be noted that owing to the long distance to the DOD-348 water molecule (and therefore a poor H bond from it), the proton of Tyr-34 does not stay exactly in the phenol ring plane, but turns partly towards DOD-348, although less than in the deposited structure (Figs. 5b and c).

The RSZD values of DOD-348 vary quite extensively, between 0.9 and 2.0. In general, it is lowest if it donates a hydrogen bond to Tyr-166 (it always donates a H bond to the backbone O atom of His-30), especially if His-30 is in the HID state (0.9–1.5), whereas the HIP and HIE states give higher values (1.1–1.7 and 1.5–2.0). If DOD-348 instead donates a H bond to Tyr-34, it is 1.4–1.9.

Likewise, the RSZD score of DOD-352 varies between 0.6 and 1.3. This water molecule is rather close to both Nδ1 of His-30 (~3.5 Å), but it is outside the ring plane and therefore the geometry is quite poor for a H bond. It is also close to DOD-348, with a proper geometry of a H bond. We tested three different interactions of this molecule. In one, it receives a H bond from DOD-348, but its two protons do not form any H bonds. This gave rather large RSZD scores, 1.0–1.3. In the second, it instead donates a H bond to DOD-348, which gave mainly lower RSZD scores of 0.6–1.1 (but 1.3 in one case). In the third, DOD-352 donates H bonds to both DOD-348 and His-30 (which is in the HIE state). This gives low RSZD scores of 0.6–0.7.

Unfortunately, the RSZD scores of the 14 residues in the QM system compensate each other so that the average value shows a very restricted variation from 0.81 to 1.03. The best result is obtained for the RD1H, RD12H and LP1H structures and the worst for one of the RDPO structures. Thus, the experimental raw data is not accurate enough to allow us to discriminate between the various protonation states using an analysis of RSZD scores.

On the other hand, there is quite a large variation in the strain energies and they follow the net charge of the QM region. Among the structures with a net neutral charge, the RD2O structures have the lowest strain energies, 143–145 kJ/mol. Among the structures with a QM charge + 2, the LP1H structures have the lowest strain, 217–223 kJ/mol. For the structures with a net charge of 1, RD1Hb has the lowest strain energy of 186 kJ/mol. It also has the lowest average RSZD score of 0.81. Therefore, this is our best suggestion for the reduced site in chain B. The average RSZD score is much better than for the deposited structure, 1.24. The strain energy is also much lower, 198, compared to 706 kJ/mol.

In particular, our calculations indicate that the structure does not necessarily show a deprotonated Gln-143. Instead, a chemically more reasonable structure has Gln-143 doubly protonated and neutral, and the Mn-bound DOD-319 as a water molecule (Fig. 4d). The two groups are connected by a hydrogen bond of 2.06 Å (O–H). One of the protons on the water molecule forms a strong hydrogen bond to Asp-159 (1.53 Å), whereas the other does not form any H bonds but points in another direction, without making any unfavourable interaction with any other residue. This indicates that it may be rather flexible, explaining why it is not clearly seen in the structure. There are no significant difference densities around the two groups, showing that such a structure explains the nuclear density equally well as the deposited structure and it is chemically more reasonable. Again, lack of clear densities for a hydrogen atom does not prove that it is not there, only that it is more flexible or may have multiple possible conformations.

However, the structure shows a prominent positive difference density (4.3σ) rather close to Tyr-166 on the side of Oη pointing away from His-30 (Fig. 5c). This difference density is decreased if Tyr-166 is protonated on this side, e.g. 3.4σ for LP1Hc (Fig. 5b). However, this structure has an appreciably larger strain energy. Therefore, we suggest that the density is better described as a partly occupied water molecule, especially as it is rather far away from the Oη atom and out of the phenol ring plane.

### Reduced structure, chain A

**Fig. 6:**
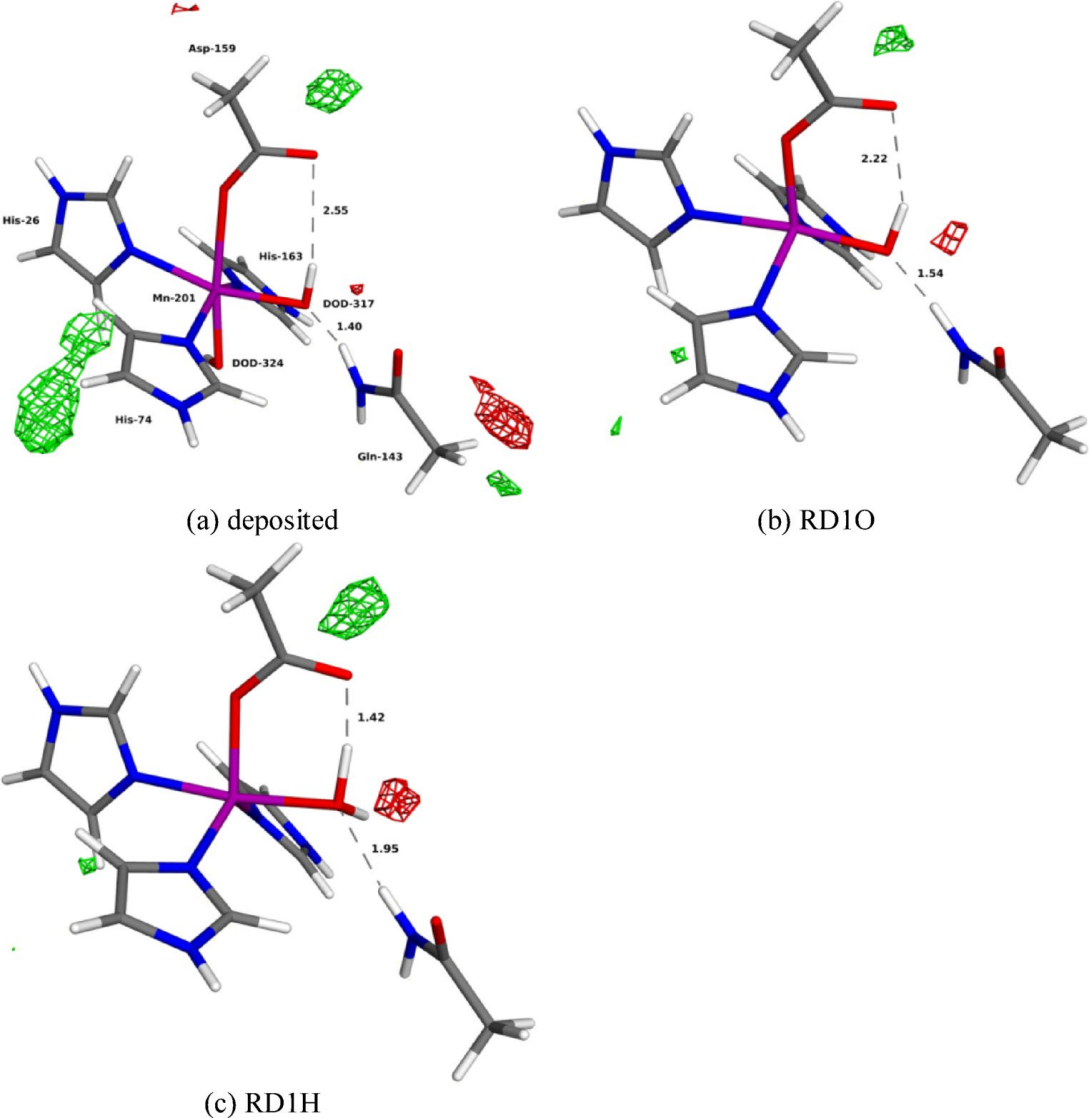
Deposited structure (2OH^–^ state) (**a**) and two QR structures in the RD1O (**b**) and RD1H (**c**) states of the Mn site of reduced MnSOD, chain A. Hydrogen bond lengths are shown in Å. The *mF*_o_ – *DF*_c_ difference maps are contoured at +3 σ (green) and –3 σ (red).

**Fig. 7:**
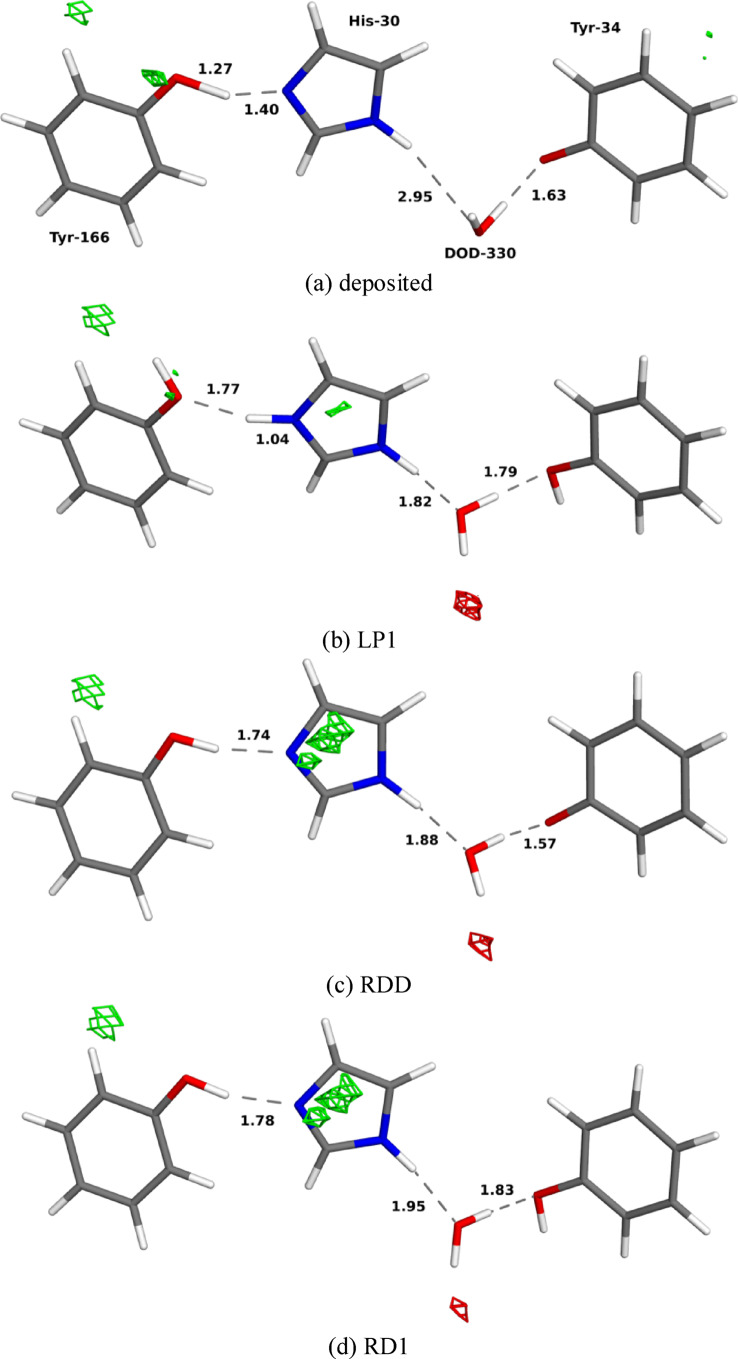
Deposited structure (RDD state) (**a**) and three QR structures of the LP1 (**b**), RDD (**c**) and RD1 (**d**) states of the H-bond chain of reduced MnSOD, chain A. Hydrogen bond lengths are shown in Å. The *mF*_o_ – *DF*_c_ difference maps are contoured at +3 σ (green) and –3 σ (red)

Finally, we turned to chain A in the reduced structure. Quite unexpectedly, the deposited structure contains two OH^–^ ligands of Mn (Fig. 6a). However, the extra OH^–^ ion is not seen in the corresponding X-ray structure and the nuclear density is much weaker for this ligand than for the other Mn ligands – there is no density overlapping with the O atom until 0.6 σ. An occupancy refinement of the occupancies of the two solvent ligands (without QR) gives occupancies of 0.97 and 0.43 (1.00 and 0.33 if ADPs are also optimised). The ADPs of the two OH^–^ molecules are ~20 and ~30 Å^2^ without the ADP optimisation and ~25 and 27 Å^2^ after optimisation, respectively. The Mn–O bond lengths are 2.12 and 1.87 Å in the neutron structure and 2.31 Å in the X-ray structure (the latter indicating water rather than OH^–^). In the deposited structure, Gln-143 is protonated and forms a very short H bond to the (first) OH^–^ Mn ligand (1.40 Å), whereas Tyr-34 is deprotonated. His-30 is protonated on Nδ1 and shares a proton with Tyr-166, although the proton is closer to Tyr (1.27 Å) than to His-30 (1.40 Å; Fig. 7a). There is a water molecule (DOD-330) between Tyr-34 and His-30, donating a strong hydrogen bond to the former (1.63 Å H–O distance), while accepting a long H bond from the latter (2.95 Å H–O distance); in fact there is a proton on the water molecule that partly points to the proton on the His-30 Nδ1 atom with a H–H distance of 2.28 Å. This water molecule is also seen in the X-ray structure.

For this site, we first tested 27 different protonation states of the hydrogen-bond chain from Tyr-166, via His-30, DOD-330 and Tyr-43 to Gln-143. They are named as in the previous sections. The results are gathered in Tables 4 and S4. The RSZD scores of Tyr-166 show only a minimal variation, 1.4–1.6, with little differences between the three tested protonation states. Likewise, the RSZD scores of Tyr-34 vary little between the various structures (1.3–1.6), with similar results for deprotonated and protonated structures with the proton either in or outside the phenol plane. The reason for this is that the nuclear density is lower on the Oη and Hη atoms of this residue (~1.1 σ) than for the other atoms in the phenol ring (2.5σ). The RSZD values of Gln-143 show a similar variation, 0.4–0.7. Structures with a protonated Tyr-34 seem to give slightly lower values, than those when it is deprotonated.

**Table 4:** Net charge of the QM system (ch), strain energies (∆*E*_str_ in kJ/mol), intrinsic QM energies (∆*E*_QM_ in kJ/mol), average RSZD scores of residues in the QM system (the individual values are shown in Table S4), as well as H-bond distances (H–O or H–N distances in Å; HB1 is between Tyr-166 and His-30, HB2 is between His-30 and DOD-330, HB3 is between DOD-330 and Tyr-34 and HB4 is between Tyr-34 and Gln-143) for the deposited structure and the various QR structures of reduced MnSOD, chain A, concentrated on the H-bond chain.

Structure	ch	∆*E*_str_	∆*E*_QM_	RSZD	HB1	HB2	HB3	HB4
deposited	-1	0	0	1.64	1.40	2.95	1.64	2.19
DED	-2	74	0	1.28	1.60	2.73	1.94	1.82
DEP	-1	106	36	1.22	1.46	2.02	1.56	2.08
DPD	-1	123	43	1.10	1.47	1.82	1.59	1.99
RDDa	-1	86	5	1.26	1.77	2.12	1.72	1.92
RDDb	-1	81	0	1.18	1.75	1.95	1.62	1.96
RDDc	-1	97	17	1.20	1.76	2.14	1.71	2.01
RDDd	-1	88	7	1.34	1.76	2.14	1.71	2.01
LED	-1	83	148	1.26	1.83	2.63	1.92	1.85
DPP	0	125	84	1.42	1.35	2.41	1.74	2.04
LPD	0	153	92	1.18	1.72	1.69	1.56	2.03
LEPa	0	63	0	1.26	1.74	1.84	1.59	2.04
LEPb	0	72	9	1.28	1.80	2.11	1.57	2.05
LE1	0	125	62	1.24	1.81	2.32	2.13	2.10
LE2	0	73	10	1.22	1.79	2.15	1.58	2.07
RDP	0	65	24	1.44	1.77	2.45	1.68	2.03
RD1a	0	54	13	1.28	1.79	2.01	1.88	2.14
RD1b	0	59	18	1.28	1.80	2.11	1.98	2.22
RD1c	0	52	11	1.36	1.80	2.15	1.92	2.19
RD2a	0	56	15	1.30	1.79	2.17	2.02	2.21
RD2b	0	62	21	1.34	1.78	2.22	2.23	2.14
RD2c	0	61	20	1.34	1.79	2.22	2.34	2.20
LPP	1	90	42	1.32	1.63	2.13	1.82	2.06
LP1a	1	51	3	1.22	1.65	1.74	1.81	2.22
LP1b	1	69	21	1.14	1.67	1.84	1.94	2.25
LP1c	1	48	0	1.24	1.66	1.78	1.84	2.22
LP2a	1	52	4	1.26	1.65	1.80	1.82	2.62
LP2b	1	74	26	1.22	1.64	1.93	1.98	2.42
LP2c	1	58	10	1.32	1.67	1.82	2.00	2.52

The RSZD scores of His-30 are lowest if only Nε2 is protonated (1.4–1.7) or if both Nε2 and Nδ1 are protonated (1.4–1.8), whereas they are larger if only Nδ1 is protonated (1.7–2.1). The RSZD values of DOD-330 show the largest variation. Structures in which it donates a hydrogen bond to Tyr-34 and accepts a hydrogen bond from His-30 give the best results, but also a very large variation (0.4–1.6), showing that it depends strongly on the direction of the second proton. Structures where it does not donate any hydrogen bonds (but accepts hydrogen bonds from both His-30 and Tyr-34) give the worst results (1.6–2.2). The other two combinations give intermediate results (1.0–1.6).

Taking all five residues together, the variation of the average RSZD score is small, 1.1–1.4. The DPD structure gives the lowest average (1.10). However, this structure can only be obtained with a restraint on the proton on Nε2 (otherwise, it is transferred to Tyr-166, giving the RDD structure). RDDb also gives a low average score (1.18) as does one of the LP1 structures (1.14). The DPP and RDP structures give the largest average RSZD score (mainly owing to the poor score for DOD-330).

Among the structures with a net charge of −1 for the QM region, the RDDb structure is intrinsically most stable. It gives the fourth-lowest average RSZD score, but it has a rather high strain energy, 81 kJ/mol. Among the structures with a neutral QM region, LEPa is intrinsically most stable, but the average RSZD score is intermediate, 1.26. The strain energy is also intermediate, 63 kJ/mol. However, the H atom on DOD-330 pointing towards His-30 is not at all supported by the nuclear density. The RD1 structures are ~11 kJ/mol less stable and have higher average RSZD scores, ~1.3, but the strain energies are lower, 52–59 kJ/mol. The LP1 structures, with a net charge of +1, give the lowest strain energies, 48–69 kJ/mol and also give among the lowest RSZD scores, 1.14–1.24 (but the one with the lowest RSZD has the largest strain energy). The average RSZD score for the RD1 and LP1 structures is better than for the deposited structure, 1.2–1.3 compared to 1.6. The strain energies are also much lower, 51–69 kJ/mol, compared to 289 kJ/mol. Consequently, our two best structures are RD1 and LP1.

Finally, we merged the two QM regions to a large one, involving ~100 atoms from both the Mn site and the H-bond chain (Figure S1d). We tested the five best interpretations of the H-bond chain (DPD, RDD, LEP, RD1 and LP1) and the three interpretations of the Mn ligands ((OH^–^)_2_, OH^–^ or H_2_O, for which we append 2, O or W to the abbreviations of the H-bond-chain models). In three of the H_2_O structures, one proton moves from H_2_O to Asp-159. This was avoided by adding a restraint on this bond (1.11 Å, the bond length in the two structures, in which the proton did not move). Structures with the proton on H_2_O or on Asp-159 differ by only 5–9 kJ/mol in energy for LEPW and RD1W, but by 103 kJ/mol for LP1W.

The results in Tables 5 and S5 show that there are quite small variations among the 15 models. The strain energies are always largest for the (OH^–^)_2_ models. For the DPD, RDD and LEP models, they are lower for the OH^–^ model than for the H_2_O model, but for RD1 and LP1, the opposite is true, although the difference is only 2 kJ/mol. The strain energies are slightly lower for the RDD and LEP models than for the other three models.

**Table 5:** Net charge of the QM system (ch), strain energies (∆*E*_str_ in kJ/mol), average RSZD scores of residues in the QM system (the individual values are shown in Table S5), as well as H-bond distances (H–O or H–N distances in Å; HB1 is between Tyr-166 and His-30, HB2 is between His-30 and DOD-330, HB3 is between DOD-330 and Tyr-34, HB4 is between Tyr-34 and Gln-143, HB5 is between Gln-143 and DOD-324, and HB6 is between DOD-324 and Asp-159) and Mn–X distances (in Å; N1–N3 are the binding N atoms of His-26, 74 and 163, whereas O1–O3 are the O atoms of Asp-159, DOD-317 and DOD-324, respectively) for the deposited structure and the various QR structures of reduced MnSOD, chain A, with a QM region including both the Mn site and the H-bond chain.

Structure		ch	∆*E*_str_	RSZD	HB1	HB2	HB3	HB4	HB5	HB6	Mn–X distance					
											N1	N2	N3	O1	O2	O3
RDD	2OH^–^	-2	307	1.21	1.76	2.16	1.67	2.05	1.58	2.18	2.20	2.22	2.29	2.26	1.98	2.06
DPD	2OH^–^	-2	352	1.22	1.74	1.94	1.58	2.06	1.57	2.18	2.20	2.22	2.29	2.26	1.97	2.06
	OH^–^	-1	101	1.21	1.75	1.89	1.59	1.98	1.63	2.19	2.22	2.25	2.24	2.04	1.94	
	HOH	0	117	1.30	1.78	1.95	2.37	1.81	2.03	1.47	2.12	2.17	2.24	2.06	2.10	
RDD	2OH^–^	-2	355	1.20	1.75	1.92	1.57	2.09	1.56	2.21	2.20	2.24	2.29	2.25	1.97	2.05
	OH^–^	-1	82	1.18	1.75	1.88	1.57	2.00	1.63	2.22	2.21	2.25	2.25	2.04	1.95	
	HOH	0	113	1.26	1.78	1.93	1.61	1.83	2.04	1.46	2.11	2.18	2.25	2.05	2.09	
LEP	2OH^–^	-1	233	1.22	1.74	1.80	1.67	2.23	1.58	2.17	2.19	2.23	2.32	2.18	1.95	2.03
	OH^–^	0	84	1.17	1.76	1.80	1.64	2.14	1.61	2.23	2.21	2.24	2.27	2.02	1.96	
	HOH	2	99	1.28	1.74	1.85	1.63	2.01	2.02	1.10	2.11	2.18	2.27	2.04	2.10	
RD10	2OH^–^	-1	257	1.26	1.73	1.98	1.84	2.20	1.46	1.43	2.22	2.21	2.29	2.25	1.99	2.1
	OH^–^	0	103	1.20	1.78	1.96	1.83	2.16	1.54	2.14	2.20	2.25	2.26	2.01	1.97	
	HOH	1	101	1.27	1.8	1.94	1.87	2.08	1.87	2.22	2.15	2.18	2.24	2.12	2.02	
LP10	2OH^–^	0	301	1.10	1.89	1.98	1.85	2.13	1.67	1.59	2.13	2.24	2.36	2.05	1.88	1.95
	OH^–^	1	96	1.08	1.77	1.81	1.79	2.19	1.66	1.42	2.16	2.22	2.29	1.98	1.92	
	HOH	2	94	1.21	1.69	1.80	1.82	2.13	1.87	2.06	2.15	2.16	2.29	2.10	2.00	

The average RSZD scores show a very small variation, 1.1–1.3. The best result is obtained for the LP1O model. It gives the lowest RSZD scores for His-30, His-74, Gln-143, Asp-159, Mn and the Mn-bond OH^–^ group (DOD-317). However, it gives among the highest RSZD scores for Tyr-166 (1.9), seen also in the difference map as significant positive density around the Oη atom (Fig. 7b). The only other significant difference density is seen in the maps near the OD1 atom of Asp-159 for all structures (least for LP1O), but especially for structures with a H_2_O ligand (and also when the proton has moved from H_2_O to Asp-159; cf. Figures 6b and c). In conclusion, it is rather hard to decide which model fits the experimental data best, probably because it contains a mixture of several states (a lower fraction with two OH^–^ ligands and a larger fraction of the RD1W model, also seen in chain B).

## Conclusions

We have performed a thorough QR study of two neutron structures of MnSOD in the oxidised and reduced states, with an emphasis of the Mn sites and residues with unusual protonation states in the vicinity (Gln-143, Tyr-34, His-30 and Tyr-166). Our approach is to do QR for all putative protonation states and use crystallographic (RSZD scores and difference maps) and QM quality measures (strain energies) to decide which structure fits the experimental data best.

By this approach, we provide alternative interpretations of both chains in the two neutron structures. For chain A of the oxidised structure, we suggest that Tyr-34 is protonated (RD1), in variance to the suggestion of a deprotonated phenolic oxygen in the deposited structure. The same applies to chain B of the same structure, but we also suggest that His-30 is protonated on the Nδ1 atom, in contrast to the observation of a doubly deprotonated imidazolate group in the deposited structure (but judged unlikely in the article [2]). For chain B of the reduced enzyme, the deposited structure suggests that the Mn-ligand is water, Gln-143 is a deprotonated CONH^–^ group and that a proton is shared between Tyr-166 and His-30. The QR results agree that the Mn-ligand is water, but Gln-143 is protonated and neutral and Tyr-166 is protonated, forming a H bond to His-30 (which has a proton on the Nδ1 atom). For the A-chain of the reduced structure, the deposited structure shows two OH^–^ ligands of Mn, a deprotonated Tyr-34 and an almost shared proton between Tyr-166 and His-30. The QR calculations indicate that this site is disordered, with a weakly occupied extra OH^–^ ligand which distorts the structure and makes it hard to interpret. However, our best interpretation is that the structure is mainly the same as in chain B, i.e. with a single water ligand, a protonated Tyr-34 and a proton on Tyr-166.

An important finding is that the phenolic Hη proton on Tyr-34 prefers to reside within the plane of the phenol ring by ~20 kJ/mol. This applies to all our preferred QR structures and contrasts to the position in chain B of the deposited reduced structure (the only structure with a protonated Tyr-34), in which the proton is distinctly out of the plane, pointing towards a nearby water molecule (for Tyr-166, the proton is always within the phenol plane). When in the phenol plane, the proton of Tyr-34 may point in two different directions (towards CE1 or CE2, respectively), without forming any H bond. We suggest that both these two conformations are occupied in the crystal structures, explaining why there is no clear nuclear density for this proton.

We provide similar explanations for several of the suggested missing protons in the deposited structures (e.g. for HOH-306 in chain A of the oxidised structure), i.e. that the proton is not seen simply because it may occupy several different positions with similar preferences or shows extensive dynamics. This is probably the most important take-home message from this study: Missing nuclear densities do not necessarily prove that the proton is not there – it may as well be disordered or flexible.

With QR, we did not find support for any of the suggested shared protons. This is expected: At the QM level, protons are very seldom perfectly shared (except when the donor and acceptor groups are identical). However, there may be two positions of the proton (on the acceptor or on the donor) with similar energies, leading to occupation of both positions and possibly extensive dynamics between the two structures, especially at room temperature. This may give an average structure that indicates a shared proton, but would require refinement with two alternative conformations in QR (not tested). Thus, a shortcoming with QR is that it cannot model dynamics within a single structure. Still, our results indicate that for the present neutron structures, there are no clear indications of shared protons. Localised protons, as in our QR structures, reproduce the experimental data equally well.

It should be emphasized that we do not claim that our structural interpretations fit the data much better than the original ones, even if our best QR structures always give better average RSZD scores than the deposited structures (and of course also much lower strain energies). We only claim that our interpretations are at least as good as the original ones and therefore that there is no strong experimental evidence for the unusual protonation states (deprotonated Tyr-34, His-30 or Gln-143). In fact, essentially none of the discussed differences in the RSZD scores is statistically significant (only RSZD scores larger than 3 are considered statistically significant [30], so it is possible that we have discussed differences partly caused by noise. It is also only few differences between the structures that are seen in the nuclear difference maps at the 3 σ level.

It should also be pointed out that the present structures show some seemingly random positions, in particular in the location of the water molecule(s) between Tyr-34 and His-30. The structure is different between the two neutron structures and the two chains in each structure, but they also differ between the neutron and X-ray structures. Moreover, the water molecule(s) often show poor H bonds. This region would probably gain from further studies with multiple crystal structures and also QR with larger QM regions to include also putative H bond partners of the water molecule(s).

Finally, from a technical point of view, we have observed several problems with the strain energies for these systems. First, as has been observed before [6], strain energies are not really comparable for systems with different net charge of the QM region. Second, the reference structure (the QM-optimised structure) typically needs to be optimised with some atoms restrained, to ensure that groups do not move too much in a way that is not possible in the protein. This has also been observed before and the normal solution is to fix atoms where a covalent bond to the protein is cleaved [18]. However, this means that water molecules may still move freely in the QM-optimised structures and sometimes give rise to large strain energies, if better H-bond partners are available somewhere in the structure, especially charged groups. Moreover, moving water molecules will always disfavour models in which the water molecules have several unsatisfied H bonds within the QM region. Third, there are often proton transfers within the QM region in the reference calculations, which may change one model to another. We have tried to employ the same protonation state in both the QR and reference QM calculation, but this is tedious and often requires the addition of restraints with unknown or arbitrary target distances. Moreover, we occasionally observe larger changes in the structure, e.g. coordination of a deprotonated Tyr residue to Mn. Thus, with many polar groups and many possible local minima, it becomes harder to define proper reference structures and strain energies may become a less accurate quality measure.

In conclusion, we think that this study nicely shows how QR can be used to interpret neutron structures and how many refinements can be run with varying protonation states to decide what structural interpretation fits the experimental data best. We think that such an approach should always be used when experimental structures suggest chemically unexpected structures or protonation states.

## Supplementary Information

Below is the link to the electronic supplementary material.

Supplementary Material 1

Supplementary Material 2

## Data Availability

Coordinates of all quantum-refined structures are provided in the supplementary material (file Coord.zip which contains the quantum region in PDB format; coordinates outside this region are not modified in the quantum refinement).
