# Supplementary material for: Critical evaluation of two neutron structures of Mn superoxide dismutase with quantum refinement
Source: J Biol Inorg Chem. 2026 Mar 10;31(3):173–91. doi: 10.1007/s00775-026-02140-5 (PMC13287255; doi:10.1007/s00775-026-02140-5)

Critical evaluation of two neutron structures of Mn superoxide dismutase with quantum refinement

Kristoffer J. M. Lundgren, Justin Bergmann, Esko Oksanen and Ulf Ryde *

Division of Computational Chemistry, Lund University, Chemical Centre, P. O. Box 124, SE-221 00 Lund, Sweden

Correspondence to Ulf Ryde, E-mail: Ulf.Ryde@compchem.lu.se,

Tel: +46 – 46 2224502

2025-10-30

Table S1. RSZD scores of residues in the QM system, as well as R and R_{free} factors for the deposited structure and the various QR structures of oxidised MnSOD, chain A. The upper part of the table contains the initial 16 QR calculations, whereas the lower part contains the subsequent eight QR calculations.

Structure	RSZD					R	R_{free}
	H30	Y34	Q143	Y166	HOH306		
deposited	1.6	1.3	0.9	4.4	1.6	0.2497	0.3010
DDD	2.4	1.1	0.2	3.7	4.6	0.2302	0.2878
DEDa	1.1	0.9	0.1	1.5	1.7	0.2297	0.2876
DEDb	1.1	1.2	0.2	1.6	1.8	0.2297	0.2871
DEP	1.1	1.0	0.1	1.5	2.4	0.2297	0.2877
LED	0.7	1.0	0.2	1.0	1.6	0.2293	0.2878
RDD	0.6	1.0	0.1	0.7	2.2	0.2296	0.2878
DPD	0.9	1.0	0.1	1.4	2.4	0.2296	0.2878
RMP	1.0	1.0	0.1	0.7	2.4	0.2293	0.2870
LEP	1.0	1.0	0.1	0.9	2.2	0.2294	0.2871
LPD	0.4	1.0	0.1	0.8	2.3	0.2293	0.2875
RDP	1.1	1.1	0.1	0.7	2.1	0.2297	0.2874
DPP	0.5	1.1	0.1	1.2	2.2	0.2299	0.2877
LPP	0.5	1.0	0.1	0.8	2.1	0.2296	0.2881
RMD	0.9	1.0	0.2	0.6	1.9	0.2297	0.2881
RM1	1.0	0.9	0.1	0.7	1.8	0.2295	0.2872
RM2	1.1	1.0	0.1	0.7	1.4	0.2299	0.2876
RDPa	0.8	1.0	0.1	0.7	2.8	0.2295	0.2871
RDPb	1.0	0.9	0.1	0.5	1.5	0.2300	0.2869
RD1a	0.9	1.0	0.2	0.7	1.8	0.2293	0.2870
RD1b	0.5	1.0	0.1	0.6	2.3	0.2291	0.2876
RD2	0.9	1.0	0.2	0.8	2.0	0.2298	0.2875

Table S2. RSZD scores of residues in the QM system, as well as R and R_{free} factors for the deposited structure and the various QR structures of oxidised MnSOD, chain B.

Structure	RSZD						R	R_{free}
	H30	Y34	Q143	Y166	HOH336	HOH347		
deposited	0.9	0.5	1.1	1.5	0.6	1.1	0.2562	0.2835
DMD	0.6	0.2	1.1	1.2	0.8	0.6	0.2303	0.2894
DED	1.3	0.4	1.2	0.5	0.9	0.5	0.2300	0.2883
RMD	0.9	0.1	1.1	0.7	0.8	0.6	0.2301	0.2878
DED	1.2	0.4	1.1	0.5	0.8	0.5	0.2302	0.2885
DEP	1.1	0.2	1.2	0.6	0.6	0.9	0.2303	0.2886
LED	1.0	0.2	1.3	0.8	0.8	0.6	0.2300	0.2881
RDD	0.7	0.4	1.2	0.6	0.8	0.4	0.2300	0.2884
RMP	0.7	0.3	1.2	0.5	0.5	0.8	0.2302	0.2880
LEP	1.0	0.4	1.4	0.6	0.8	1.1	0.2301	0.2881
RDP	0.8	0.3	1.1	0.5	0.6	1.3	0.2302	0.2880
RD1a	0.8	0.1	1.1	0.7	0.6	0.7	0.2301	0.2886
RD1b	0.7	0.2	1.2	0.6	2.3	0.5	0.2300	0.2884
RD1c	0.8	0.2	1.1	0.7	2.2	0.9	0.2301	0.2883
RD1d	0.8	0.5	1.2	0.7	0.5	0.7	0.2302	0.2881
RD1e	0.9	0.2	1.0	0.8	1.2	0.9	0.2303	0.2878
RD2a	0.9	0.2	1.1	0.6	0.6	0.7	0.2297	0.2883
RD2b	0.7	0.6	0.9	0.7	1.8	0.6	0.2303	0.2875
RD2c	0.7	0.3	1.0	0.6	1.2	1.2	0.2299	0.2883
RD2d	0.8	0.4	1.1	0.4	0.4	0.8	0.2303	0.2878
RD2e	0.8	0.1	1.0	0.7	1.2	0.8	0.2298	0.2887

Table S3. RSZD scores of residues in the QM system, as well as R and R_{free} factors for the deposited structure and the various QR structures of reduced MnSOD, chain B.

Structure	RSZD														R	R_{free}
	H 26	H 30	H 31	Y 34	H 74	W 123	Q 143	D 159	H 163	Y 166	Mn	DOD 319	DOD 348	DOD 352		
deposited	0.7	0.9	1.6	0.5	1.0	1.8	1.7	0.7	2.5	1.0	0.9	1.7	1.2	1.1	0.2496	0.3009
DPDW	0.2	0.4	1.2	1.6	0.4	1.2	0.3	0.8	0.9	0.9	1.2	1.1	1.4	1.2	0.2268	0.3033
RDDW	0.4	0.4	1.2	1.8	0.5	1.5	0.3	0.8	0.6	0.8	1.3	1.1	1.5	1.2	0.2264	0.3035
RDPOa	0.4	0.9	1.4	1.2	0.4	1.5	0.6	0.7	0.8	0.8	1.3	1.2	1.4	1.2	0.2264	0.3036
RDPOb	0.3	1.0	1.3	1.3	0.4	1.4	0.5	0.7	1.0	1.1	1.4	1.4	1.6	1.0	0.2265	0.3032
RD1Oa	0.2	0.6	1.3	1.0	0.2	1.5	0.4	0.8	0.7	1.1	1.4	1.0	1.0	0.8	0.2263	0.3035
RD1Ob	0.3	0.4	1.4	1.0	0.2	1.4	0.4	0.8	0.8	1.2	1.5	1.0	1.5	1.0	0.2259	0.3027
RD1Oc	0.3	0.3	1.0	1.1	0.3	1.5	0.4	0.7	0.8	1.2	1.5	1.1	1.3	0.7	0.2264	0.3025
RD2Oa	0.2	0.6	1.0	1.1	0.4	1.4	0.4	0.7	0.8	0.9	1.5	1.1	1.2	0.8	0.2263	0.3030
RD2Ob	0.4	0.5	1.0	1.1	0.4	1.6	0.5	0.8	0.9	1.0	1.5	1.1	1.1	0.8	0.2265	0.3034
RD2Oc	0.3	0.4	1.0	1.1	0.4	1.6	0.5	0.7	0.8	1.0	1.4	1.1	1.3	0.7	0.2266	0.3029
RDDW	0.3	1.0	1.2	1.7	0.5	1.3	0.3	0.8	0.8	1.1	1.3	1.1	1.5	1.2	0.2264	0.3035
LPDW	0.2	0.9	1.3	1.5	0.5	1.1	0.5	0.8	0.9	0.6	1.4	1.1	1.5	1.3	0.2264	0.3033
LPPO	0.3	0.8	1.4	1.1	0.5	1.4	0.4	0.8	0.9	0.6	1.2	1.2	1.5	1.3	0.2262	0.3029
LPPO	0.2	0.9	1.3	1.1	0.4	1.2	0.4	0.8	1.0	0.5	1.3	1.2	1.7	1.3	0.2261	0.3035
RDPH	0.3	1.2	1.4	1.4	0.4	1.3	0.2	1.0	0.8	1.2	0.7	1.2	1.9	1.2	0.2265	0.3033
RD1Ha	0.2	1.1	0.9	1.0	0.2	1.3	0.1	0.9	0.8	1.2	0.6	1.4	0.9	0.9	0.2263	0.3026
RD1Hb	0.2	0.7	1.0	1.1	0.2	1.3	0.1	1.0	0.8	1.2	0.6	1.1	0.9	1.1	0.2261	0.3035
RD1Hc	0.3	0.5	1.1	1.1	0.3	1.5	0.1	0.9	0.8	1.2	0.6	1.2	1.2	0.7	0.2261	0.3015
RD2Ha	0.3	1.0	1.3	1.2	0.4	1.4	0.1	0.8	0.7	1.1	0.7	1.0	1.2	0.9	0.2265	0.3025
RD2Hb	0.2	0.7	1.0	1.3	0.4	1.3	0.1	0.9	0.8	1.1	0.6	1.1	1.2	0.9	0.2260	0.3029
RD2Hc	0.3	0.6	0.9	1.1	0.4	1.5	0.2	0.8	0.8	1.1	0.5	1.2	1.5	0.8	0.2263	0.3029
LE1H	0.3	1.2	1.4	1.1	0.5	1.4	0.3	0.9	0.6	0.5	0.6	1.1	2.0	0.7	0.2260	0.3031
LE2H	0.3	2.1	1.3	1.3	0.5	1.3	0.1	0.7	0.6	0.5	0.6	1.2	1.5	0.6	0.2264	0.3023
LPPH	0.3	1.2	1.3	1.3	0.4	1.3	0.2	1.1	0.9	0.7	0.7	1.1	1.7	1.2	0.2261	0.3026
LP1Ha	0.2	1.1	1.4	1.0	0.2	1.2	0.1	1.0	0.9	0.6	0.6	1.4	1.1	0.8	0.2261	0.3020
LP1Hb	0.3	0.9	1.3	0.9	0.3	1.5	0.1	0.9	0.8	0.6	0.6	1.3	1.2	1.3	0.2262	0.3028
LP1Hc	0.3	0.9	0.9	1.0	0.3	1.3	0.1	0.9	0.9	0.6	0.6	1.4	1.5	0.6	0.2264	0.3016
LP2Ha	0.2	1.1	1.2	1.3	0.4	1.5	0.1	0.8	0.8	0.5	0.7	1.2	1.6	1.0	0.2264	0.3037
LP2Hb	0.3	1.0	1.3	1.2	0.4	1.3	0.2	1.1	0.8	0.6	0.7	1.5	1.4	0.8	0.2268	0.3040
LP2Hc	0.2	0.9	1.4	1.2	0.4	1.4	0.1	0.9	0.8	0.5	0.7	1.3	1.7	0.7	0.2267	0.3034

Table S4. RSZD scores of residues in the QM system, as well as R and R_{free} factors for the deposited structure and the various QR structures of reduced MnSOD, chain A, concentrated on the H-bond chain.

Structure	RSZD					R	R_{free}
	H30	Y34	Q143	Y166	DOD330		
deposited	0.9	1.4	1.7	1.9	2.3	0.2496	0.3009
DED	1.4	1.4	0.6	1.4	1.6	0.2265	0.3043
DEP	1.7	1.4	0.4	1.4	1.2	0.2261	0.3030
DPD	1.4	1.4	0.7	1.5	0.5	0.2263	0.3030
RDDa	2.1	1.3	0.5	1.5	0.9	0.2263	0.3029
RDDb	1.9	1.4	0.6	1.6	0.4	0.2262	0.3032
RDDc	2.0	1.4	0.4	1.5	0.7	0.2260	0.3031
RDDd	2.1	1.4	0.6	1.5	1.1	0.2264	0.3030
LED	1.5	1.3	0.6	1.6	1.3	0.2260	0.3035
DPP	1.7	1.5	0.5	1.5	1.9	0.2262	0.3028
LPD	1.8	1.4	0.6	1.5	0.6	0.2260	0.3031
LEPa	1.7	1.4	0.5	1.5	1.2	0.2260	0.3034
LEPb	1.7	1.3	0.6	1.4	1.4	0.2260	0.3028
LE1	1.6	1.4	0.4	1.4	1.4	0.2256	0.3037
LE2	1.7	1.4	0.6	1.5	0.9	0.2257	0.3027
RDP	1.7	1.4	0.4	1.5	2.2	0.2261	0.3025
RD10	1.8	1.5	0.4	1.5	1.2	0.2264	0.3029
RD11	1.8	1.5	0.4	1.5	1.2	0.2259	0.3032
RD12	1.7	1.3	0.5	1.6	1.7	0.2264	0.3024
RD20	1.8	1.5	0.4	1.5	1.3	0.2259	0.3029
RD21	2.0	1.5	0.5	1.5	1.2	0.2262	0.3027
RD22	1.7	1.3	0.5	1.5	1.7	0.2257	0.3029
LPP	1.7	1.4	0.5	1.6	1.4	0.2259	0.3025
LP10	1.7	1.4	0.7	1.5	0.8	0.2269	0.3027
LP11	1.6	1.4	0.5	1.4	0.8	0.2260	0.3025
LP12	1.7	1.4	0.7	1.4	1.0	0.2265	0.3021
LP20	1.7	1.5	0.6	1.5	1.0	0.2259	0.3021
LP21	1.7	1.6	0.5	1.4	0.9	0.2259	0.3023
LP22	1.6	1.4	0.6	1.5	1.5	0.2257	0.3024

Table S5. RSZD scores of residues in the QM system, as well as R and R_{free} factors for the deposited structure and the various QR structures of reduced MnSOD, chain A, with a QM region including both the Mn site and the H-bond chain.

Structure		RSZD													R	R_{free}
		H 26	H 30	Y 34	H 74	W 123	Q 143	D 159	H 163	Y 166	Mn	DOD 317	DOD 324	DOD 330		
RDD	2OH	0.2	1.5	1.4	2.1	0.7	0.7	1.3	1.2	1.6	0.7	2.0	1.5	0.8	0.2263	0.3035
DPD	2OH	0.2	2.0	1.5	2.1	0.8	0.7	1.3	1.1	1.5	0.6	2.0	1.5	0.6	0.2263	0.3037
	OH	0.3	2.0	1.3	2.0	0.8	0.8	1.3	1.2	1.5	0.5	2.1		0.7	0.2254	0.3040
	HOH	0.3	2.0	1.4	2.0	0.7	0.7	1.9	1.3	1.5	0.7	2.5		0.6	0.2251	0.3051
RDD	2OH	0.2	1.9	1.4	2.2	0.7	0.6	1.2	1.1	1.5	0.6	2.1	1.5	0.6	0.2260	0.3040
	OH	0.2	1.9	1.3	2.1	0.8	0.7	1.3	1.2	1.5	0.4	2.2		0.6	0.2252	0.3044
	HOH	0.2	1.9	1.3	2.0	0.7	0.7	1.7	1.2	1.6	0.7	2.5		0.6	0.2248	0.3049
LEP	2OH	0.2	1.4	1.4	2.3	0.8	0.7	1.3	1.2	1.6	0.6	2.1	1.3	1.0	0.2268	0.3044
	OH	0.2	1.3	1.2	2.0	0.9	0.7	1.3	1.2	1.5	0.4	2.1		1.2	0.2254	0.3039
	H2O	0.2	1.3	1.4	2.2	0.8	0.6	1.8	1.1	1.5	0.8	2.5		1.2	0.2250	0.3045
RD10	2OH	0.2	1.7	1.5	2.1	0.8	0.7	1.3	1.3	1.5	0.5	2.5	1.6	0.7	0.2264	0.3044
	OH	0.2	1.7	1.4	2.0	0.9	0.5	1.3	1.3	1.5	0.4	2.3		0.9	0.2254	0.3035
	HOH	0.3	1.6	1.3	2.0	0.8	0.5	1.9	1.2	1.6	0.9	2.2		0.9	0.2251	0.3034
LP10	2OH	0.2	1.3	1.5	2.1	0.7	0.5	1.3	1.1	1.9	0.6	1.4	0.9	0.8	0.2260	0.3042
	OH	0.3	1.2	1.4	2.1	0.8	0.5	1.2	1.0	1.9	0.3	1.4		0.9	0.2254	0.3037
	HOH	0.3	0.9	1.3	1.9	0.7	0.5	1.9	1.0	2.0	0.9	2.1		1.0	0.2251	0.3039

Figure S1. The quantum systems used in the various QR calculations: (a) oxidised MnSOD chain A, (b) oxidised MnSOD chain B, (c) reduced MnSOD chain B and (d) reduced MnSOD chain A. Hydrogen bond lengths are shown in Å.

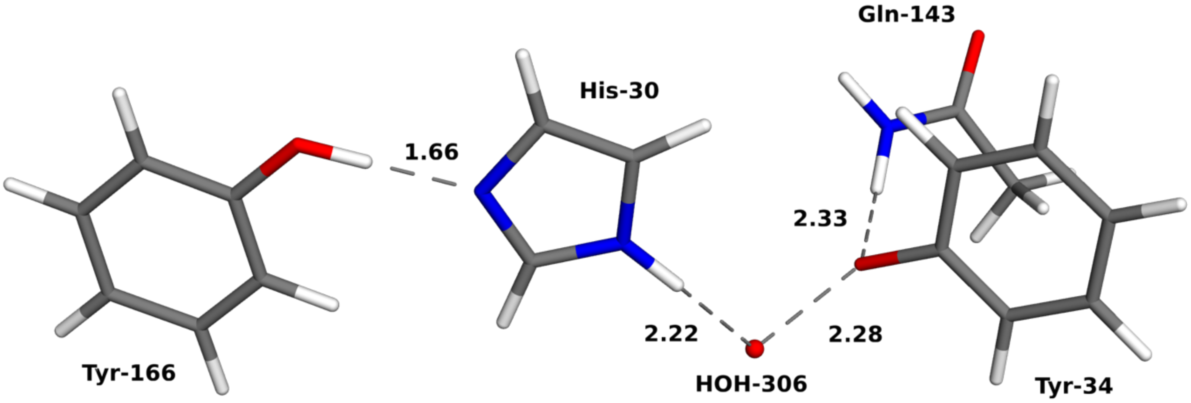

(a) oxidised MnSOD chain A

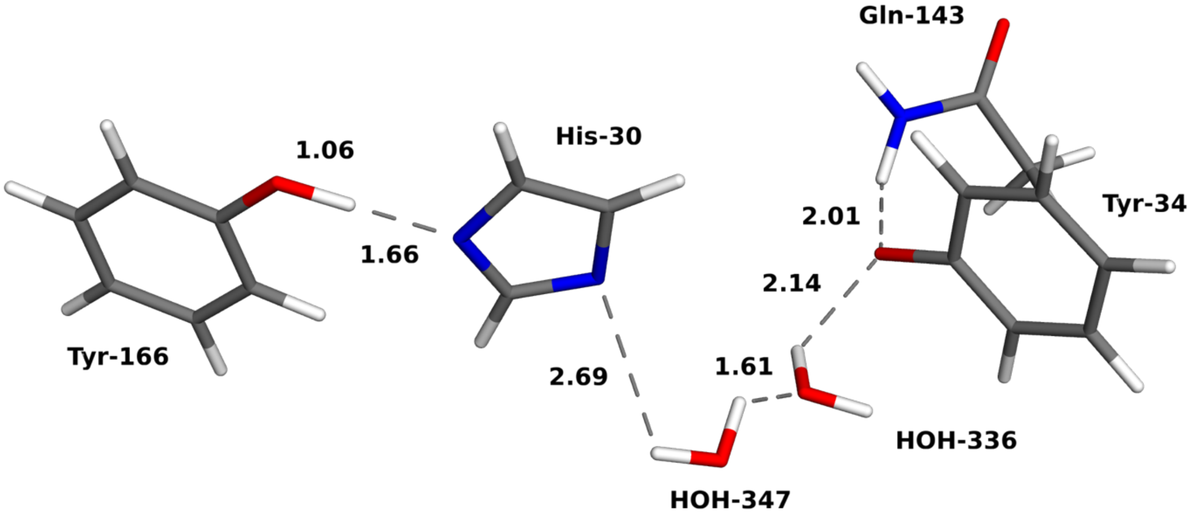

(b) oxidised MnSOD chain B

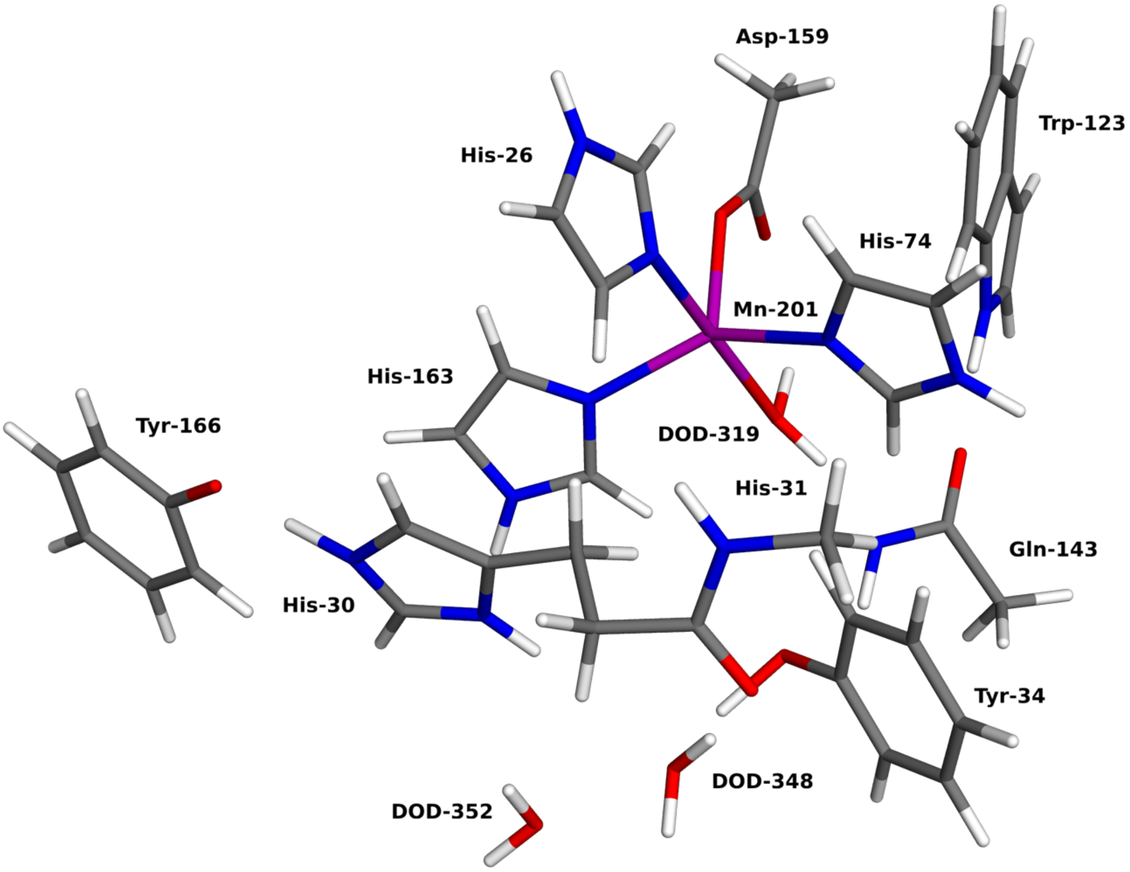

(c) reduced MnSOD chain B

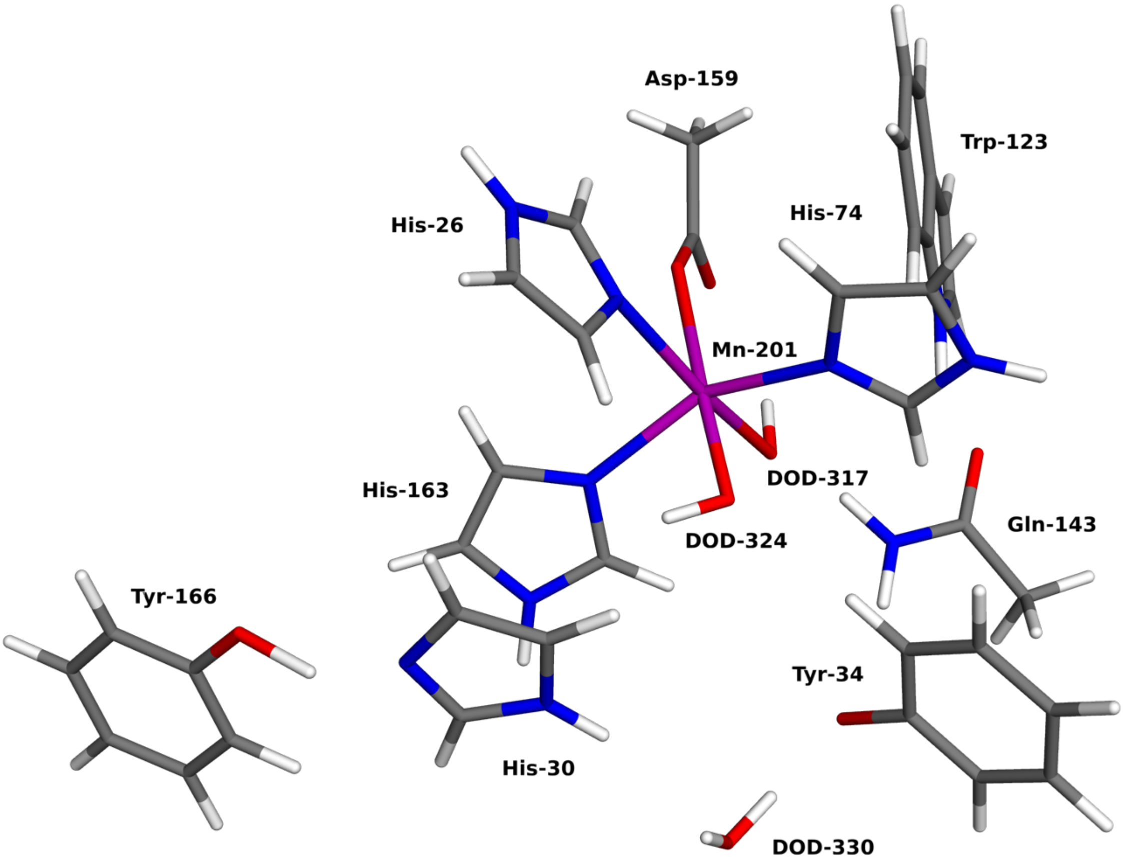

(d) reduced MnSOD chain A

Figure S2. Energy for the movement of the phenolic H atom of phenol and p-kresol out of the aromatic ring plane (C–C–O–H dihedral), calculated at the TPSS-D4/def2-SV(P) level.

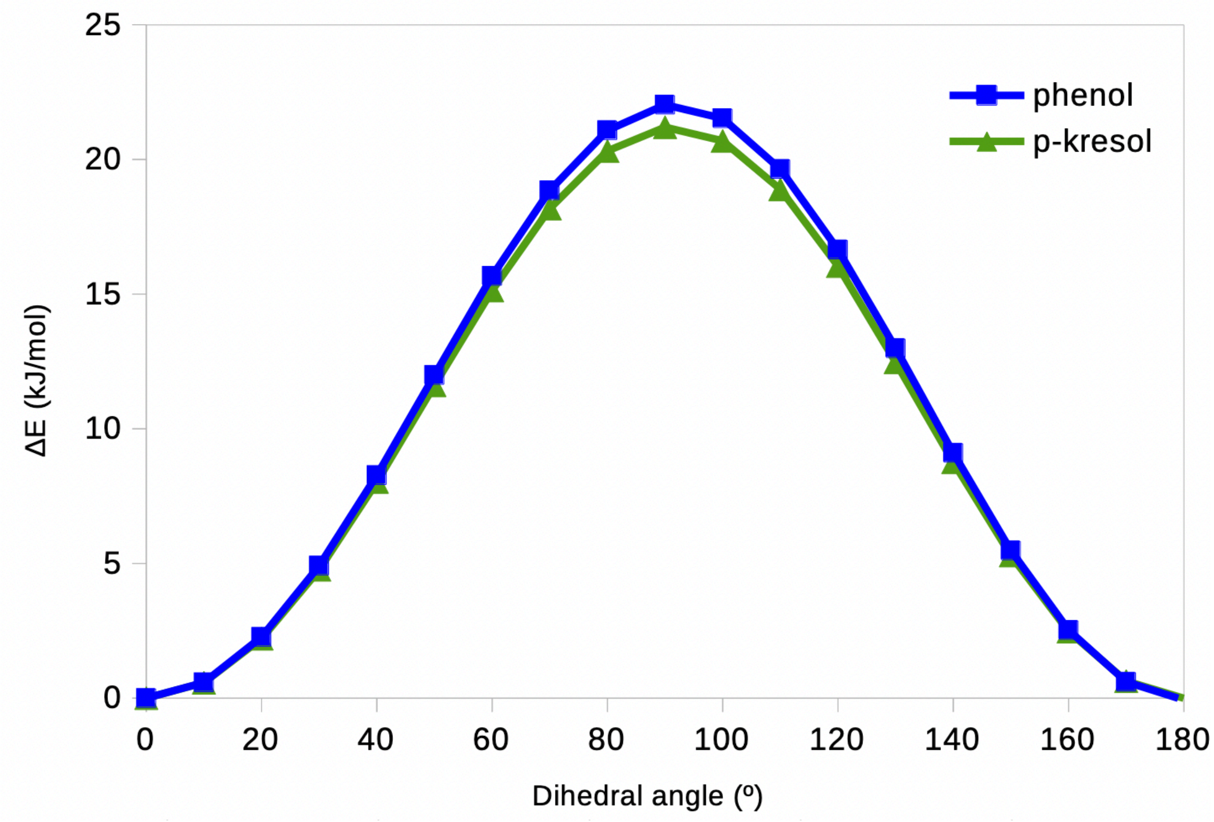

Supplement: Supplementary file 1 — Supplementary Material 1 [file 775_2026_2140_MOESM1_ESM.pdf]
